# Rapidly Inhibiting the Inflammatory Cytokine Storms and Restoring Cellular Homeostasis to Alleviate Sepsis by Blocking Pyroptosis and Mitochondrial Apoptosis Pathways

**DOI:** 10.1002/advs.202207448

**Published:** 2023-03-17

**Authors:** Jiahui Yan, Jingwen Zhang, Yanan Wang, Hong Liu, Xueping Sun, Aixin Li, Pengfei Cui, Liangmin Yu, Xuefeng Yan, Zhiyu He

**Affiliations:** ^1^ Frontiers Science Center for Deep Ocean Multispheres and Earth System and Key Laboratory of Marine Chemistry Theory and Technology Ministry of Education Ocean University of China Qingdao 266100 China; ^2^ College of Chemistry and Chemical Engineering Ocean University of China Qingdao 266100 China; ^3^ College of Marine Life Sciences Ocean University of China Qingdao 266003 China

**Keywords:** BAPTA‐AM, HMPDA, NAD^+^, pyroptosis, sepsis

## Abstract

Pyroptosis, systemic inflammation, and mitochondrial apoptosis are the three primary contributors to sepsis's multiple organ failure, the ultimate cause of high clinical mortality. Currently, the drugs under development only target a single pathogenesis, which is obviously insufficient. In this study, an acid‐responsive hollow mesoporous polydopamine (HMPDA) nanocarrier that is highly capable of carrying both the hydrophilic drug NAD^+^ and the hydrophobic drug BAPTA‐AM, with its outer layer being sealed by the inflammatory targeting peptide PEG‐LSA, is developed. Once targeted to the region of inflammation, HMPDA begins depolymerization, releasing the drugs NAD^+^ and BAPTA‐AM. Depletion of polydopamine on excessive reactive oxygen species production, promotion of ATP production and anti‐inflammation by NAD^+^ replenishment, and chelation of BAPTA (generated by BA‐AM hydrolysis) on overloaded Ca^2+^ can comprehensively block the three stages of sepsis, i.e., precisely inhibit the activation of pyroptosis pathway (NF‐*κ*B‐NLRP3‐ASC‐Casp‐1), inflammation pathway (IL‐1*β*, IL‐6, and TNF‐*α*), and mitochondrial apoptosis pathway (Bcl‐2/Bax‐Cyt‐C‐Casp‐9‐Casp‐3), thereby restoring intracellular homeostasis, saving the cells in a state of “critical survival,” further reducing LPS‐induced systemic inflammation, finally restoring the organ functions. In conclusion, the synthesis of this agent provides a simple and effective synergistic drug delivery nanosystem, which demonstrates significant therapeutic potential in a model of LPS‐induced sepsis.

## Graphical abstract

Depletion of excessive reactive oxygen species, replenishment of nicotinamide adenine dinucleotide (NAD^+^), and chelation of overloaded Ca^2+^ can comprehensively block the three stages of sepsis, saving the cells in a state of “critical survival”, further restoring the organ functions of sepsis mice.

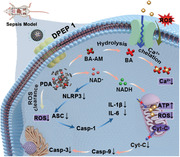

## 1 Introduction

Sepsis, a systemic inflammatory syndrome caused by immune hyperactivation and dysregulation of oxidative stress triggered by pathogenic microorganisms, eventually progresses to multiple organ dysfunction syndrome (MODS), the leading cause of death in the intensive care unit (ICU), accounting for ≈20% of deaths worldwide each year.^[^ ^1^ ^]^ More than 95% of clinical sepsis can be attributed to the release of the endotoxin lipopolysaccharide (LPS) by bacterial infections. Clinically, the treatment of sepsis is highly dependent on broad‐spectrum antibiotics and fluid resuscitation; however, the therapeutic effect is not obvious, and the mortality rate is still as high as 50%.^[^ ^2^ ^]^ Currently, hundreds of drug candidates under investigation only target a specific hit in sepsis, which is insufficient for sepsis with a complex pathogenesis; therefore, it is imperative to develop drugs that simultaneously act on multiple targets.^[^ ^3^ ^]^

Pyroptosis and apoptosis are the two most important factors that contribute to the high mortality rate in sepsis.^[^ ^4^ ^]^ When LPS invaded an organism, it first combined with Toll‐like receptor 4 (TLR4) on the membrane to activate nuclear factor kappa‐B (NF‐*κ*B), which then upregulated NOD‐like receptor thermal protein domain associated protein 3 (NLRP3) mRNA transcription.^[^ ^5^ ^]^ Oligomerization of the activated NLRP3 structural protein causes it to bind to pyridinoline (PYD) of the adapter protein apoptosis‐associated speck‐like protein containing CARD (ASC), followed by caspase recruitment domain containing protein (CARD) of ASC binding to CARD on pro‐Caspase‐1 (pro‐Casp‐1) to form a complete and active NLRP3 inflammasome, thereby promoting self‐cleavage of pro‐Casp‐1, in turn producing the active effector protein Caspase‐1 (Casp‐1).^[^ ^6^ ^]^ Activated caspase‐1, on the one hand, cleaves Gasdermin‐D (GSDMD), induces cell membrane perforation, and triggers cell scorching; on the other hand, it cleaves IL‐1*β* and IL‐18 precursors, forming active IL‐1*β* and IL‐18, which are released into the extracellular space and recruit inflammatory cells to aggregate and amplify the inflammatory response.^[^ ^7^ ^]^

The inflammatory response further activates subsequent mitochondrial apoptosis, thereby accelerating organ failure.^[^ ^8^ ^]^ Inflammation disrupts the microcirculatory system, which increases microvascular permeability, activates leukocytes, and decreases vascular resistance and blood flow, leading to tissue hypoxia and impaired perfusion.^[^ ^9^ ^]^ During hypoxia, the Na^+^‐Ca^2+^ exchanger reverse type is activated, causing a large Ca^2+^ influx into the cell.^[^ ^10^ ^]^ In addition, the accumulation of hypoxanthine during hypoxia reacts with oxygen molecules to produce a large number of reactive oxygen species (ROS), which damages the cell membrane and ER membrane, thus leading to the influx of Ca^2+^ into the cytoplasm.^[^ ^11^ ^]^ The overloaded Ca^2+^ will continue to accumulate in mitochondrial sites, resulting in irreversible over‐opening of the mitochondrial permeability transition pore (MPTP) and mitochondrial membrane potential (MMP) collapse, inhibition of ATP synthesis, mass production of ROS, and release of Cyt‐C, thereby triggering the caspase cascade reaction and cell apoptosis.^[^ ^12^ ^]^ It has been discovered that intracellular metabolic disorder such as energy deficiency, calcium overload, and ROS overproduction form a cascade reaction that reinforces each other, leading to exaggerated cell death and, eventually multiple organ failure.^[^ ^13^ ^]^ To improve the survival rate of sepsis patients, in addition to inhibiting pyroptosis, it is also necessary to prevent mitochondrial dysfunction by supplementing energy and blocking the production of excessive Ca^2+^ and ROS.

Nicotinamide adenine dinucleotide (NAD^+^), a promising anti‐inflammatory drug, inhibits the NLRP3 inflammasome pathway to reduce the production of pro‐inflammatory cytokines ^[2a]^. Moreover, NAD^+^ can also enhance the energy supply of cells and restore the vitality of damaged cells.^[^ ^14^ ^]^ NAD^+^ is a negatively charged, hydrophilic, small molecule that diffuses poorly across cell membranes.^[^ ^15^ ^]^ As a result, it is difficult for NAD^+^ to enter cells to exert anti‐inflammatory effects, which greatly reduces its biological activity and limits its clinical translational potential. As a safe and selective Ca^2+^ chelating agent, BAPTA‐AM (BA‐AM, O, O′‐bis(2‐aminophenyl) ethyleneglycol‐N,N,N′,N′‐tetraacetic acid, tetraacetoxymethyl ester) does not chelate Ca^2+^ during blood transport.^[^ ^16^ ^]^ Once it enters the cell, it can be hydrolyzed by esterase into BA, which can rapidly conjugate with Ca^2+^ to lower the intracellular Ca^2+^ concentration. Since it is a hydrophobic drug with low oral and intravenous bioavailability, its clinical use is also hampered.^[^ ^16^, ^17^ ^]^

**Scheme 1:**
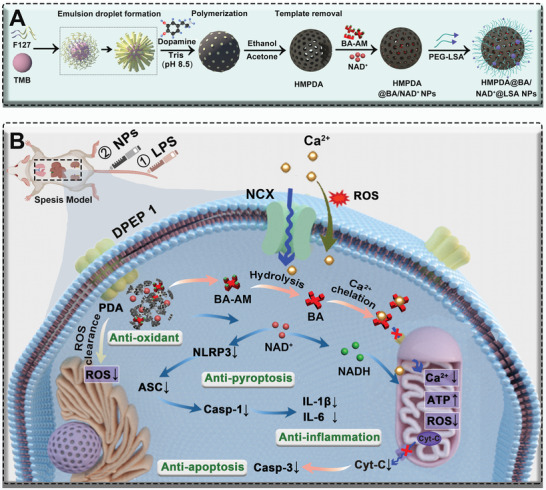
Schematic illustration of the preparation route, the sepsis animal model establishment process, and the treatment mechanism of NPs. A) Schematic diagram of preparation of HMPDA@BA/NAD^+^@LSA NPs. B) Therapeutic mechanism of BA‐AM and NAD^+^ co‐loaded NPs in an LPS‐induced sepsis mouse model.

In this study, we designed inflammation‐targeting (LSALT peptide‐modified), NAD^+^ (loading content: 50.4%) and BA‐AM (loading content: 37.4%) co‐loaded, acid‐responsive ultrasmall hollow mesopore polydopamine nanoparticles (HMPDA@BA/NAD^+^@LSA NPs, ≈70 nm) (**Scheme** **1**A). The NPs reached the injured organs by binding specifically to the highly expressed dipeptidase 1 (DPEP1) at the inflammatory site. Then, HMPDA quickly cleared ROS from the inflamed site and depolymerized in a slightly acidic inflammatory environment to release BA‐AM and NAD^+^.^[^ ^18^ ^]^ PDA's removal of ROS, BA's (generated by BA‐AM hydrolysis) chelation of overloaded Ca^2+^, and the replenishment of the NAD^+^ pool promoted the recovery of mitochondrial function, further inhibiting cell apoptosis. In addition, these effects realized anti‐oxidative stress in cells, and played an anti‐inflammatory role, thereby inhibiting cell pyroptosis. At the cellular level, we proved the in vitro biocompatibility of NPs and their protective effect on damaged cells (correction of energy metabolism, chelation of excess Ca^2+^, clearance of toxic ROS, recovery of cell viability, etc.). In the LPS‐induced sepsis mouse model, after treatment with HMPDA@BA/NAD^+^@LSA NPs (HMPDA: 320 µg kg^−1^; BA‐AM: 200 µg kg^−1^; NAD^+^: 320 µg kg^−1^), the survival rate of sepsis model mice was increased from 18.8% to 68.8%, and the level of organ edema, liver function, kidney function, and tissue oxidative stress were significantly improved, with no significant difference compared to normal mice. In addition, no significant pathological changes or apoptosis were observed in the organs of the HMPDA@BA/NAD^+^@LSA NPs treatment group (the TUNEL‐positive ratio in the liver, kidney, and lung decreased from 52.1%, 51.3%, and 59.5% to 15.2%, 13.4%, and 11.4%, respectively), indicating that the nanoagent can prevent the pathophysiological deterioration of organs and resist cell apoptosis. The HMPDA@BA/NAD^+^@LSA NPs’ therapeutic mechanism in sepsis was verified at the molecular level, that is, it simultaneously inhibited the activation of pyroptosis pathways (NF‐*κ*B, NLRP3, ASC, Casp‐1, and IL‐1*β*), inflammatory pathways (TNF‐*α* and IL‐6), and mitochondrial apoptosis pathways (Cyt‐C, Casp‐9, and Casp‐3). Overall, the drug delivery system constructed for the treatment of sepsis has achieved remarkable results, paving the way for its prospective clinical application (Scheme 1B).

## 2 Results and Discussion

### 2.1 Synthesis and Characterization of HMPDA@BA/NAD^+^@LSA NPs

**Figure 1:**
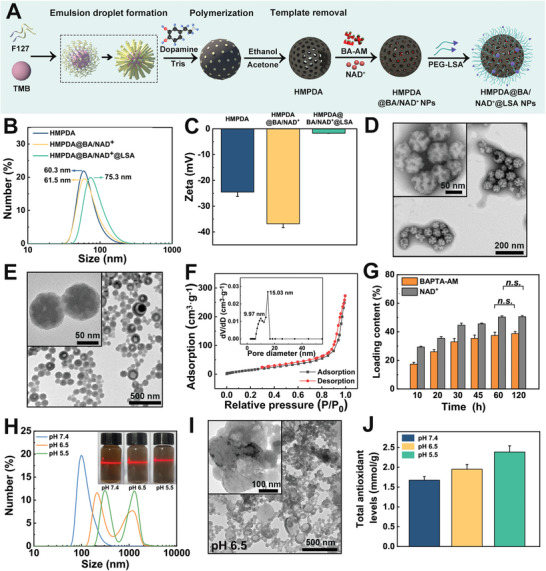
Synthesis and characterization of HMPDA@BA/NAD^+^@LSA NPs. A) Representation schematic of HMPDA@BA/NAD^+^@LSA NPs. B) Size distributions and C) *ζ*‐potential of HMPDA NPs, HMPDA@BA/NAD^+^ NPs, and HMPDA@BA/NAD^+^@LSA NPs. TEM image of D) HMPDA@BA/NAD^+^ NPs and E) HMPDA@BA/NAD^+^@LSA NPs. F) N_2_ adsorption–desorption isotherm and pore‐size distribution of HMPDA@BA/NAD^+^ NPs. G) Influence of incubation time on BA‐AM and NAD^+^ loading content (calculated by determination of free drug content) in HMPDA NPs. H) Size distributions and a photograph of the “Tyndall effect” after the response of HMPDA NPs in simulated media (PBS) at 37 °C. I) TEM image of HMPDA NPs’ responsiveness in simulated media (PBS: pH 6.5) for 1 h. J) Evaluation of ROS consumption by HMPDA (30 µg mL^−1^). Data are means ± SD, *n* = 3.

As shown in **Figure** **1**A, HMPDA was prepared using the soft template method, BA‐AM and NAD^+^ were loaded to produce HMPDA@BA/NAD^+^ NPs, and PEG‐LSA was grafted to obtain HMPDA@BA/NAD^+^@LSA NPs. Dynamic light scattering (DLS) results revealed that the hydration radius of blank HMPDA NPs was ≈60.3 nm, whereas the hydration radius of HMPDA@BA/NAD^+^@LSA NPs was no significant change (Figure 1B). However, the hydration radius of HMPDA@BA/NAD^+^ NPs grafted with PEG‐LSA increased from 61.5 to 75.3 nm, and the surface charge increased from −37.8 mV to nearly neutral (−2.1 mV), which implied that the PEG‐LSA grafting was successful (Figure 1C). In addition, the characteristic UV absorption peaks of free NH_2_‐PEG‐MAL (215 nm) and Cys‐LSA (197 nm) in the supernatant almost disappeared after grafting, further confirming the successful grafting of PEG‐LSA (Figure S1, Supporting Information). Notably, the aforementioned three NPs were uniformly dispersed in PBS (Figure S2, Supporting Information). The results of TEM revealed that the particle sizes of HMPDA and HMPDA@BA/NAD^+^@LSA were ≈50 and 70 nm, respectively (Figure 1D,E), which were slightly smaller than the hydration radius, which was conducive to the transport of NPs to the lesion site via transcytosis (10–100 nm).^[^ ^19^ ^]^ Moreover, TEM images also demonstrated that the HMPDA had numerous surface mesoporous structures and cavities, which provided sufficient space for drug loading (Figure 1D). After PEG‐LSA grafting, the pores on the HMPDA surface were covered, which was beneficial to prevent drug leakage (Figure 1E).

Brunauer–Emmett–Teller (BET) results of HMPDA and HMPDA@BA/NAD^+^ showed that the specific surface area and pore size of NPs decreased significantly after drug loading (specific surface area: from 106 to 90.5 m^2^ g^−1^; cavity: from 20.4 to 15.0 nm; channels: from 11.0 to 9.9 nm), providing evidence for the successful loading of the drug (Figure S3, Supporting Information ¸and Figure 1F). We then further evaluated the drug loading performance of the carrier by forward determination (the amount of drug successfully loaded in the HMPDA NPs) and reverse determination (the amount of free drug not successfully loaded in the HMPDA NPs) for LC and EE (Figure 1G and Figure S4, Supporting Information). When a single drug was loaded, the LC and EE of NAD^+^ and BA‐AM nearly reached saturation within 1 h. Specifically, the LC and EE of NAD^+^ in the HMPDA@NAD^+^ NPs were ≈49.3% (forward measurement: 48.2 ± 2.9%; reverse measurement: 50.4 ± 0.9%) and 19.6% (forward measurement: 18.7 ± 2.2%; reverse measurement: 20.5 ± 1.2%), respectively; the LC and EE of BA‐AM in the HMPDA@BA NPs were about 36.7% (forward measurement: 35.9 ± 0.7%; reverse measurement: 37.4 ± 2.3%) and 57.9% (forward measurement: 56.1 ± 1.6%; reverse measurement: 59.8 ± 2.1%), respectively, indicating that HMPDA could be loaded with hydrophilic or hydrophobic drugs with high efficiency in a short period of time. Surprisingly, the EE of NAD^+^ in HMPDA@BA/NAD^+^ was not affected by BA loading (the EE of NAD^+^ in HMPDA@BA/NAD^+^ NPs was 20.5 ± 1.2%, the EE of NAD^+^ in HMPDA@NAD^+^ NPs was 21.3% ± 0.9%). When the double drugs (NAD^+^ and BA‐AM) were loaded, the LC of NAD^+^ and BA‐AM in PDA@BA/NAD^+^ NPs were 38.3% and 23.8%, respectively, indicating that the carrier had excellent drug loading capacity.

Theoretically, based on the synthesis principle of PDA, which was polymerized under alkaline conditions, the carrier could undergo depolymerization and release drugs in a slightly acidic inflammatory environment.^[^ ^20^ ^]^ When HMPDA NPs were transferred from the simulated normal physiological environment (PBS, pH 7.4) to the simulated inflammatory slightly acid environment (PBS, pH 6.5), the particle size became disorderly, increasing from nanometers to microns (Figure 1H). We reasonably speculated that this was due to the depolymerization of HMPDA, which collapsed hollow mesoporous structures and triggered the aggregation of fragments. After 6 h, the NPs had visibly settled, the “Tyndall effect” light path had been interrupted, and visible solid particles were moving. The TEM image of the NPs after an acidic response (pH 6.5) showed that the HMPDA structure was destroyed and aggregation occurred, confirming our hypothesis (Figure 1I). In the medium (PBS) with pH of 7.4, 6.5, and 5.5, the cumulative drug release of HMPDA@BA/NAD^+^@LSA NPs at 24 h were 9.4%, 41.6%, and 47.9% for BA and 10.1%, 57.6%, and 67.7% for NAD^+^, respectively, indicating that under weak acid conditions, the rupture of nanocarriers was beneficial to the rapid release of drugs in the pathological microenvironment (Figure S5, Supporting Information). At a physiological pH environment, the release rate of the two drugs from HMPDA@BA/NAD^+^@LSA NPs was less than 10% within 48 h, demonstrating that the nanomedicine can effectively avoid drug leakage in systemic circulation, thereby reducing the toxicity and adverse effects on normal tissue. As shown in Figure 1J, HMPDA has exceptional antioxidant capacity, and as acidity increases, its antioxidant capacity gradually increases. At pH 5.5, the antioxidant capacity of HMPDA was 1.4 times that at pH 7.4. This is primarily because the structure of HMPDA contains a large number of phenolic hydroxyl groups, and the acidic environment serves to amplify the antioxidant activities, making it favorable for HMPDA to consume ROS in the slightly acidic inflammatory environment. Within a week, the particle size of the NPs remained around 75 nm and the PDI remained around 0.12 without significant changes, indicating that the NPs were stable at RT for an extended period of time, which is favorable for future clinical use (Figure S6, Supporting Information).

### 2.2 Cell Study

#### 2.2.1 In Vitro Biocompatibility of NPs

**Figure 2:**
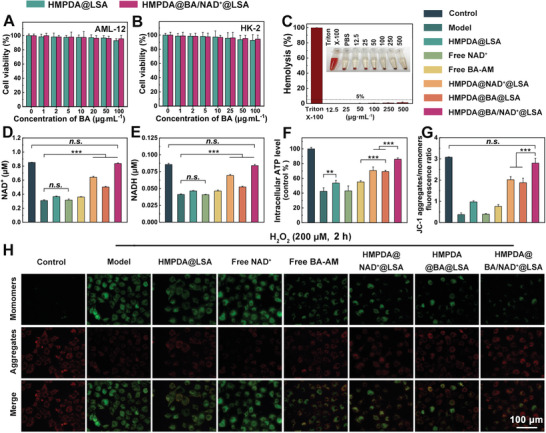
Restoration of cellular energy homeostasis. Cell viability (CCK‐8) of A) AML‐12 cells and B) HK‐2 cells was incubated with different doses (based on BA‐AM) of HMPDA@LSA NPs and HMPDA@BA/NAD^+^@LSA NPs for 24 h. C) Relative hemolysis ratios of various concentrations of HMPDA@BA/NAD^+^@LSA NPs. The pretreatment process of the following experiments is consistent, and the specific operation is as follows: H_2_O_2_‐stimulated AML‐12 cells were incubated with PBS, HMPDA@LSA (HMPDA: 320 ng mL^−1^), free NAD^+^ (320 ng mL^−1^), free BA‐AM (200 ng mL^−1^), HMPDA@NAD^+^@LSA (NAD^+^: 320 ng mL^−1^), HMPDA@BA@LSA (BA‐AM: 200 ng mL^−1^), HMPDA@BA/NAD^+^@LSA (BA‐AM: 200 ng mL^−1^, NAD^+^: 320 ng mL^−1^) for 12 h, respectively. D) NAD^+^, E) NADH, and F) ATP levels in H_2_O_2_‐stimulated AML‐12 cells with different treatment. G) Semiquantitative fluorescence results and H) fluorescence images of the JC‐1 assay to measure mitochondrial membrane depolarization in H_2_O_2_‐stimulated AML‐12 cells after different treatments. JC‐1 is a probe for detecting mitochondrial membrane potential. JC‐1 “aggregates” at a higher mitochondrial membrane potential (red fluorescence, normal state). At a lower mitochondrial membrane potential, JC‐1 become “monomers” (green fluorescence, apoptotic state). Data are mean ± SD, *n* = 6.

As shown in **Figure** **2**A,B, blank HMPDA@LSA NPs and HMPDA@BA/NAD^+^@LSA NPs exhibited no cytotoxic effects on AML‐12 and HK‐2 cells at concentrations ranging (based BA‐AM concentration) from 1 to 100 µg mL^−1^. In addition, to ensure the safety of NPs in the bloodstream, hemolysis experiments were conducted. As shown in Figure 2C, even when the concentration of HMPDA@BA/NAD^+^@LSA NPs (based on HMPDA concentration) was as high as 500 µg mL^−1^, there was no obvious hemolysis phenomenon, and the hemolysis rate was significantly lower than the threshold value (5%), indicating that the nanodrug was safe for blood circulation.

#### 2.2.2 Restoration of Intracellular Energy Homeostasis

The production of large amounts of ATP involves three stages. The first stage is glycolysis, which occurs in the cytoplasm, where two NAD^+^ molecules are converted by glucose into two NADH molecules, and the glucose is broken down to produce pyruvate, which is transported to the mitochondria.^[^ ^21^ ^]^ The second stage occurs in the mitochondrial matrix, where pyruvate consumes NAD^+^ molecules and generates an equal amount of NADH.^[^ ^22^ ^]^ The third stage is the mitochondrial respiratory chain, which occurs in the inner membrane of the mitochondria, where NADH molecules produced by the tricarboxylic acid cycle are oxidized into NAD^+^ by coenzyme Q10 and a large amount of ATP is simultaneously produced [Equation (5)].^[^ ^23^ ^]^

(1) $$\begin{array}{c} \mathrm{NADH}+H^{+}+\mathrm{CoQ}+4H^{+}\mathrm{in}\to {\mathrm{NAD}}^{+}+{\mathrm{CoQH}}_{2}+4H^{+}\mathrm{out} \end{array}$$

When sepsis occurs, respiratory action within the attacked cell is disrupted, the energy metabolism pathway of the mitochondria is destroyed, and the basic cellular activities are unable to be maintained.^[^ ^24^ ^]^ Here, we used H_2_O_2_‐stimulated AML‐12 cells as an in vitro model of acute liver cell injury and then treated the injured cells with different prescriptions for 12 hours to determine the effect of NPs on mitochondrial respiratory pathways. As illustrated in Figure 2D, the content of NAD^+^ in AML‐12 cells induced by H_2_O_2_ decreased by 62.2% relative to normal cells. Compared with the model group, the level of NAD^+^ in the HMPDA@LSA NPs group, the free BA‐AM group, and the HMPDA@BA@LSA group increased by 18.3%, 16.3%, and 66.7%, respectively. This may be because HMPDA and BA eliminated the excess ROS and Ca^2+^ in the damaged cells, respectively, prevented further mitochondrial damage in the cells, and induced the gradual production of endogenous NAD^+^. However, the intracellular NAD^+^ content did not increase after treatment with free NAD^+^, which may be because free NAD^+^ is a hydrophilic drug that cannot easily traverse the cell membrane. In the HMPDA@NAD^+^@LSA NPs group, the intracellular NAD^+^ level was about two times that of the model group, indicating that HMPDA could efficiently transport NAD^+^ into cells while eliminating ROS. Surprisingly, the level of NAD^+^ in the cell of the HMPDA@BA/NAD^+^@LSA NPs group was close to that of normal cell, which further confirmed that the NAD^+^ library could not only be “injected” through exogenous replenishment but also promote the generation of endogenous NAD^+^ by restoring mitochondrial function.

In addition, the rise in intracellular NAD^+^ further leads to an increase in intracellular NADH levels.^[2a]^ As shown in Figure 2E, NADH content in injured cells after treatment increased by 75.3% in the HMPDA@NAD^+^@LSA group compared to the model group, while NADH content in the HMPDA@BA/NAD^+^@LSA group was close to normal cells, which was consistent with NAD^+^ results. The NAD^+^/NADH ratio is another important indicator of cell metabolism and redox state. We compared the changes in NAD^+^/NADH values in each group since the higher the ratio, the better the cell state.^[^ ^25^ ^]^ We found that the NAD^+^/NADH ratio of damaged cells was about 75.2% that of normal cells, and that after treatment with HMPDA@LSA and HMPDA@NAD^+^@LSA, the NAD^+^/NADH ratio of model cells returned to 79.0% and 90.3% of control cells, respectively. Encouragingly, NAD^+^/NADH levels even returned to normal levels after HMPDA@BA/NAD^+^@LSA NPs treatment, further demonstrating that NAD^+^ supplementation, ROS clearance, and Ca^2+^ chelation can synergistically restore intracellular oxidative stress homeostasis (Figure S7, Supporting Information).

To verify the recovery effect of NAD^+^ replenishment on the mitochondrial respiratory chain, the intracellular ATP and MMP levels were measured. As shown in Figure 2F, compared to the level of ATP in the damaged cells, the ATP content in the HMPDA@NAD^+^@LSA NPs group was significantly increased, reaching 72.3% of that in the control cells, demonstrating that the replacement of NAD^+^ may further stimulate the production of ATP. As expected, the ATP level of the HMPDA@BA/NAD^+^@LSA NPs group was twofold higher than that of the model group and was close to normal, indirectly confirming the importance of ROS and Ca^2+^ clearance for the recovery of mitochondrial function. MMP is an intuitive indicator of normal mitochondrial activity. Generally, the higher the value, the more energy is generated. As shown in Figure 2G,H, the MMP of AML‐12 cells induced by H_2_O_2_ was reduced to around 12% of its normal level. MMP in the HMPDA@LSA NPs group was almost twofold higher than in the model group, indicating that elimination of excess ROS could prevent mitochondrial dysfunction to some extent. The MMP in the HMPDA@NAD^+^@LSA NPs group and the HMPDA@BA@LSA NPs group were all ≈5 times that of the model group, exhibiting that NAD^+^ replenishment and Ca^2+^ chelation could help mitochondria recover their normal function. The MMP of cells treated with dual‐drug co‐loaded NPs was significantly higher than that of cells treated with single‐drug loaded NPs but did not differ significantly from the control group. The survival rate of AML‐12 cells after injury after treatment with double‐drug loaded NPs also reached 93.2% (Figure S8, Supporting Information). These findings revealed that NAD^+^ replenishment and inhibition of mitochondrial damage can maximally restore mitochondrial function, regulate energy homeostasis, and save dying cells.

#### 2.2.3 Cellular Protective Effect of HMPDA@BA/NAD^+^@LSA NPs

**Figure 3:**
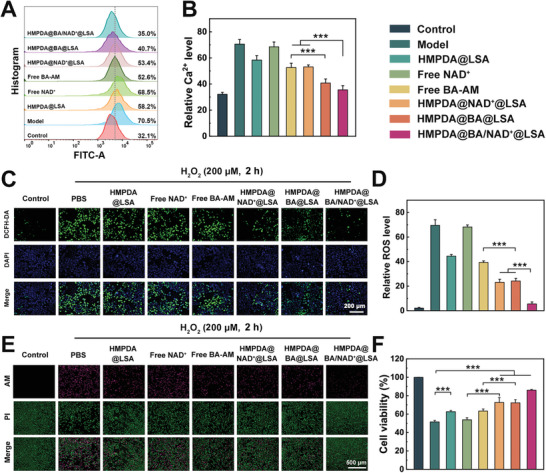
Cellular protective effect of HMPDA@BA/NAD^+^@LSA NPs. The pretreatment process for the following experiments is consistent, and the specific steps are as follows: H_2_O_2_‐stimulated HK‐2 cells were incubated with PBS, HMPDA@LSA (HMPDA: 320 ng mL^−1^), free NAD^+^ (320 ng mL^−1^), free BA‐AM (200 ng mL^−1^), HMPDA@NAD^+^@LSA (NAD^+^: 320 ng mL^−1^), HMPDA@BA@LSA (BA‐AM: 200 ng mL^−1^), HMPDA@BA/NAD^+^@LSA (BA‐AM: 200 ng mL^−1^, NAD^+^: 320 ng mL^−1^) for 12 h, respectively. A) The alteration of Ca^2+^ level in the H_2_O_2_‐stimulated HK‐2 cell line with different treatments was determined by flow cytometry. B) Quantitative flow cytometry results for the relative Ca^2+^ level. C) Fluorescence images, and D) semiquantitative results of intracellular ROS. E) The cell viability of H_2_O_2_‐induced acutely injured HK‐2 cells after 12 h of treatment. F) Fluorescence images and G) semiquantitative results of AM/PI‐stained injured HK‐2 cells after various treatments. Data are mean ± SD, *n* = 6.

To further verify the above findings, we used H_2_O_2_‐stimulated HK‐2 cells as in vitro models of acute cell injury to determine the effects of NPs on Ca^2+^ levels, ROS levels, and cell necrosis levels. As depicted in **Figure** **3**A,B, after H_2_O_2_ stimulation, the Ca^2+^ levels increased dramatically, almost twofold in comparison to normal cells, indicating that intracellular calcium homeostasis was destroyed. The Ca^2+^ concentration decreased by nearly 20% after treatment with blank carrier (HMPDA@LSA NPs), due to the PDA's elimination of ROS in time, further blocking Ca^2+^ influx. The scavenging effect of NAD^+^‐loaded NPs was significantly greater than that of the blank carrier group, so we speculated that this was because the restoration of mitochondrial vitality improved the mitochondria's capacity to recycle Ca^2+^. Unsurprisingly, the HMPDA@BA/NAD^+^@LSA NPs group reduced intracellular relative Ca^2+^ levels by ≈50.3% compared to the model group, returning to levels close to those of the control group. This significant finding indicated that HMPDA@BA/NAD^+^@LSA NPs have a protective effect on cells by promoting calcium homeostasis recovery, that is, by directly chelating Ca^2+^ and indirectly inhibiting Ca^2+^ inflow via a synergistic action that eliminates Ca^2+^ overload.

In addition, intracellular ROS levels in injured cells were directly measured to evaluate their antioxidant capacity (Figure 3C,D). Compared to the model group, the ROS level in the blank carrier group was reduced by 38.4%, indicating that HMPDA itself can rapidly consume excess intracellular ROS. The anti‐ROS effect of HMPDA@ NAD^+^@LSA NPs (decline of 64.3%) was significantly better than that of a blank carrier, as the replenishment of NAD^+^ could prompt cells to consume oxygen and generate ATP, thereby reducing the possibility of ROS production. Notably, the level of ROS in the HMPDA@BA@LSA NPs group was reduced by 61.4%, because BA‐AM could indirectly inhibit the production of ROS by chelating Ca^2+^ and simultaneously play an anti‐ROS role with HMPDA. Notably, the HMPDA@BA/NAD^+^@LSA NPs treatment group had the highest ROS consumption capacity, and the relative ROS level of cells was reduced by about 90.4%, corresponding to the Ca^2+^ level, indicating that the simultaneous replenishment of NAD^+^ repertory, anti‐oxidation, and Ca^2+^ chelation could synergistically eliminate ROS and relieve Ca^2+^ overload.

To verify whether NPs can rescue injured cells, we assessed the cell viability of HK‐2 cells treated with different NPs after H_2_O_2_ stimulation. Compared to the model group, HMPDA@NAD^+^@LSA NPs and HMPDA@BA@LSA NPs significantly reversed cell viability (cell viability: increased from 43.8% to 69.8% and 70.8%, respectively) (Figure S8, Supporting Information). Specifically, the cell viability of the HMPDA@BA/NAD^+^@LSA NPs treatment group was even higher than 80%, approaching that of the control cell, highlighting the advantages of the three‐pronged intervention. Through live/dead cell labeling, the salvage effect of NPs on damaged cells will be visualized more clearly. As shown in Figure 3E,F, the cell survival rates after treatment with HMPDA@BA@LSA NPs and HMPDA@NAD^+^@LSA NPs were both higher than 75%, confirming that in addition to scavenging ROS, replenishing the NAD^+^ pool or chelating Ca^2+^ could restore cell viability. Consistent with the above cell viability assay, the cell survival rate of the HMPDA@BA/NAD^+^@LSA NPs group was up to 80% compared to the large number of dead cells in the model group (Figure S9, Supporting Information). In summary, the NPs could replenish the intracellular NAD^+^ pool, remove excess ROS and Ca^2+^, and thus reduce intracellular oxidative stress, restore intracellular calcium homeostasis, recover normal mitochondrial function, and ultimately save dying cells.

### 2.3 LPS‐Induced Sepsis Mice Treatment

**Figure 4:**
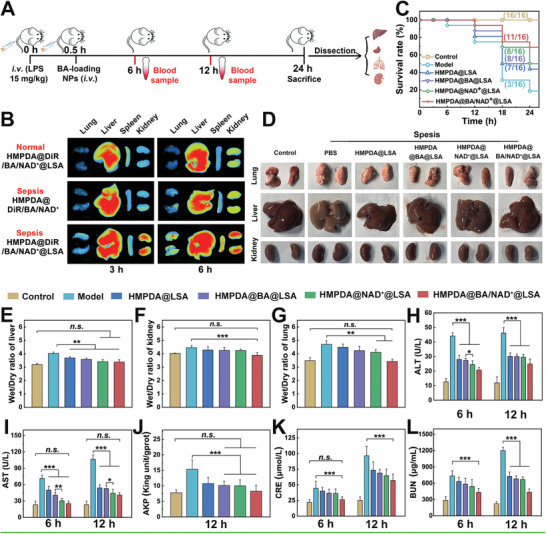
In vivo therapeutic effect in mice sepsis model. A) Experimental flowchart of the treatment process. B) Ex vivo NIR imaging of major organs (heart, liver, spleen, lungs, and kidneys) in the sepsis mice. Data are mean ± SD, *n* = 3. C) Survival rate of sepsis mice within 1 d after different treatments. Data are mean ± SD, *n* = 16. D) Photos of major organs of each group after dissection. The wet/dry ratio of E) liver, F) kidney, and G) lung in sepsis mice from each group. H,I) Blood serum ALT and AST levels from each group, respectively. J) AKP levels of liver tissue in different groups. K,L) Blood serum CRE and BUN levels from each group, respectively. Data are mean ± SD, *n* = 6.

To better mimic the clinical practice requirements of sepsis, we chose LPS‐induced (15 mg kg^−1^) mice as the sepsis animal model. The treatment procedure was depicted as the timeline in **Figure** **4**A. To explore the biodistribution in vivo, the formulation loading DiR near‐infrared probe was injected immediately after LPS injection, and the distribution of NPs in major organs was observed by an in vitro imaging system 3 and 6 h after administration. Moreover, the distribution of NPs in healthy mice was observed for comparison purposes. As shown in Figure 4B and Figure S10 (Supporting Information), the accumulation of NPs (with targeted peptides) in the liver, kidney, and lung of sepsis model mice was 1.3, 2.7, and 2.3 times that of control mice at 3 h, respectively, and continued to accumulate in the liver, lungs, and kidneys of sepsis mice and started to accumulate in the spleen at 6 hours. The accumulation of NPs with targeted peptide in the kidney and lung of sepsis‐model mice was 2 and 1.7 times that of NPs without targeted peptide at 3 h after injection, respectively. At 6 h, the accumulation of NPs with targeted peptide in the liver, kidney, and lung of sepsis mice was 1.4, 1.6, and 1.5 times higher than that NPs without targeted peptide. These results indicated that LSA can assist HMPDA@BA/NAD^+^@LSA NPs to rapidly target and accumulate in the damaged sites with high DPEP1 expression to exert therapeutic effect and further prevent sepsis from exacerbating into systemic organ failure, which seems to have greater clinical value.

To evaluate the therapeutic effect of NPs, survival rates of sepsis mice treated with different NPs were measured (Figure 4C). Within 24 h, the mortality rate for sepsis model mice was up to 80%, whereas the mortality rate for sepsis mice treated with HMPDA@LSA NPs, HMPDA@BA@LSA NPs and HMPDA@NAD^+^@LSA NPs was 56.3%, 50.0%, and 50.0%, respectively. Surprisingly, the mortality rate of sepsis mice treated with HMPDA@BA/NAD^+^@LSA NPs was about 30%, suggesting that the nanoagent could potentially save sepsis mice in critical condition. In addition, the liver, kidney, and lung organs of the sepsis mice were slightly larger than those of the control group (Figure 4D). By measuring the wet‐dry weight ratios of liver, kidney, and lung organs in different groups, it was found that the organs of the sepsis model mice did have the edema phenomenon (Figure 4E–G). However, the organs of mice treated with HMPDA@BA/NAD^+^@LSA NPs reverted to those of control mice.

The therapeutic effect of the nanoagent on the liver and kidney was then evaluated by measuring key markers in serum and tissue. After 6 h of modeling, serum ALT and AST levels in the model group nearly quadrupled in comparison to the control group (Figure 4H,I). However, at 6 and 12 h after treatment with HMPDA@NAD^+^@LSA NPs, ALT decreased by 44.5% and 33.3% and AST decreased by 60.1% and 57.1%, respectively. In addition, 12 h following treatment with HMPDA@NAD^+^@LSA NPs, ALT and AST levels were significantly lower than those in the HMPDA@BA@LSA NPs group (*p* < 0.05). We reasonably speculated that NAD^+^ has multiple effects that can not only inhibit inflammatory storms directly but also provide NAD^+^ for the mitochondrial respiratory chain, thereby restoring energy supply. BAPTA‐AM suppressed the production of ROS induced by Ca^2+^ overload only by chelating Ca^2+^, which can effectively prevent the further deterioration of the disease with a relatively solitary and slow effect. Expressly, there was no significant difference in AST level between HMPDA@BA/NAD^+^@LSA NPs group and control group at 6 and 12 h after treatment. The AKP content in the liver tissue of the HMPDA@BA/NAD^+^@LSA NPs group was nearly 50% lower than that of the model group, no different from that of control mice (Figure 4J). The decreased levels of ALT, AST, and AKP indicated that the liver function of the treated mice had returned to normal.

CRE and BUN are the main indicators of renal function that can be used to assess renal excretory function.^[^ ^26^ ^]^ As shown in Figure 4K,L, 12 h after modeling, the levels of CRE and BUN in the model group were approximately fourfold and sixfold higher than that in the control group, respectively, indicating renal impairment. Compared to the model group, in the HMPDA@NAD^+^@LSA NPs group, CRE and BUN decreased by 32.3% and 41.7%, respectively, which were better than the treatment effect of HMPDA@BA@LSA NPs. Furthermore, in the HMPDA@BA/NAD^+^@LSA NPS group, the levels of CRE and BUN were reduced by 45.3% and 66.7%, respectively, suggesting that the NPs themselves had a beneficial effect on alleviating kidney injury. The above data indicated that our formulation greatly increased the survival rate of sepsis mice and promoted the normalization of liver and kidney function. It is therefore reasonable to assume that the formulation avoided further cell damage by eliminating ROS and Ca^2+^ from the damaged cells. Meanwhile, the replenishment of NAD^+^ provided energy to the damaged cells, resulting in the rapid restoration of organ function.

**Figure 5:**
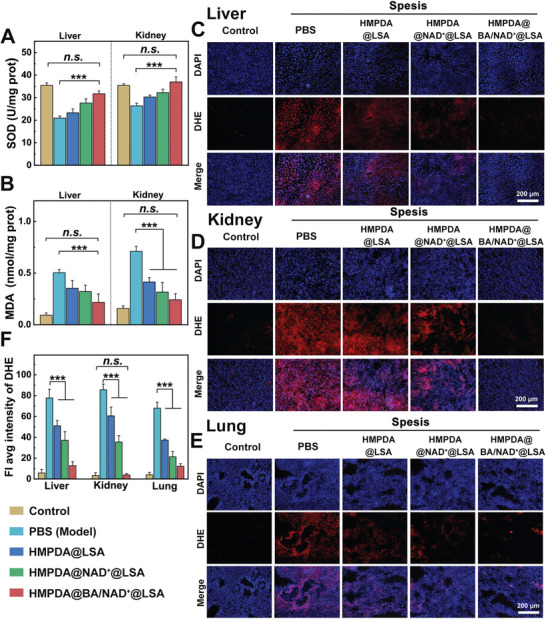
In vivo therapeutic effect in the sepsis mouse model. A) Liver tissue SOD, and B) MDA concentrations for different groups in the sepsis animal model. Dihydroethidium (DHE) staining of C) liver, D) kidney, and E) lung tissue in different groups (red fluorescence: DHE; blue fluorescence: cell nucleus). F) DHE fluorescence semiquantitative results in different groups. Data are mean ± SD, *n* = 6.

Oxidative stress caused by high levels of ROS is one of the important mechanisms of organ damage in sepsis. To further verify the antioxidant effect of NPs in vivo, we evaluated the levels of two major oxidative stress markers (SOD and MDA) in injured liver and kidney tissue. As shown in **Figure** **5**A, SOD levels in liver and kidney tissue in the model group were reduced by nearly 42.9% and 28.6%, respectively, compared to those of the control group, indicating that the damaged liver and kidney had a diminished ability to scavenge free radicals. After treatment with HMPDA@BA/NAD^+^@LSA NPs, the SOD content in liver and kidney tissue was ≈1.5 and 1.4 times that of the model group, respectively, with no significant difference from that of the control group, indicating that the antioxidant system of the kidney was restored to normal. MDA is an indicator that can reflect the degree of lipid peroxidation, which can accumulate in large quantities under ROS oxidation, thus aggravating cell damage. MDA levels in the liver and kidney of the model group were higher (nearly five and four times those of the control group, respectively), and MDA levels after treatment with HMPDA@BA/NAD^+^@LSA NPs were reduced by about 60% and 67%, respectively, which did not differ significantly from the control group, indicating that lipid peroxidation in the damaged liver and kidney tissues was inhibited (Figure 5B). To further validate the antioxidant effect of NPs in vivo, DHE staining was performed on fresh liver, kidney, and lung tissues to assess the global condition of oxidative stress after treatment (Figure 5C–F). Compared with the model group, DHE fluorescence intensity (red fluorescence) in the liver, kidney, and lung of the HMPDA@BA/NAD^+^@LSA NPs treatment group was reduced by 83.8%, 94.1%, and 84.3%, respectively, indicating that this preparation can alleviate multi‐organ oxidative damage caused by sepsis and create space for the recovery of organ function.

We believe that HMPDA@BA/NAD^+^@LSA NPs can restore oxidative stress homeostasis primarily due to the three factors listed below: 1) the rapid clearance of ROS by PDA, thereby preventing organ damage caused by ROS; 2) the replenishment of NAD^+^, which increases the body's oxygen consumption and ATP production, thus reducing the possibility of ROS production; and 3) the quick chelation of Ca^2+^ by BA can restore intracellular Ca^2+^ homeostasis and prevent the increase of ROS caused by Ca^2+^ overload.

**Figure 6:**
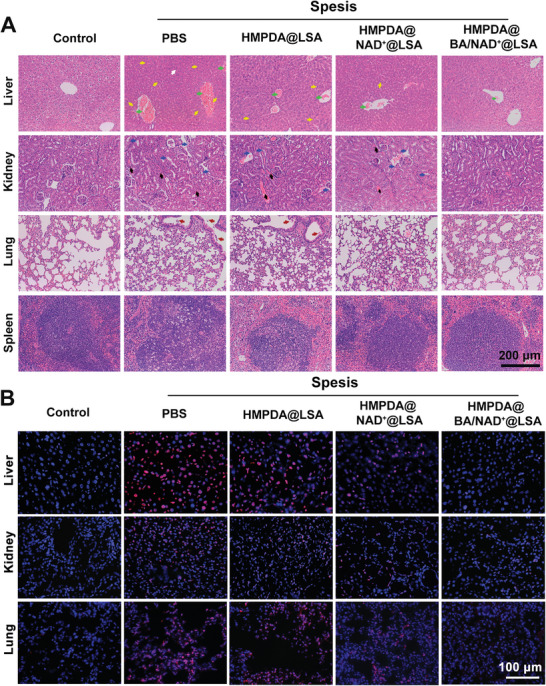
In vivo therapeutic effect in the sepsis mice model. A) H&E staining of liver, kidney, lung, and spleen tissue. Green arrows indicate central hepatic vein ischemia; yellow arrows indicate liver necrosis cell; white arrows indicate inflammatory cell infiltration; blue arrows indicate renal tubular epithelial cell exfoliation site; black arrows indicate necrotic shedding of renal tubular epithelial cells to form casts; and red arrows indicate accumulation of neutrophils. B) TUNEL staining of liver, kidney, and lung tissue (red fluorescence: TUNEL positive cells; blue fluorescence: cell nucleus). Data are mean ± SD, *n* = 6.

To more intuitively evaluate the therapeutic effect of HMPDA@BA/NAD^+^@LSA NPs, H&E staining was performed to observe the improvement of the pathophysiology of the organs (liver, kidney, lung, and spleen) (**Figure** **6**A). Compared to healthy mice, the pathological features of the model mouse's liver included inflammatory cell infiltration (white arrow), central venous engorgement (green arrow), and hepatocyte necrosis (yellow arrow). In contrast, after treatment with HMPDA@BA/NAD^+^@LSA NPs, there was only a slight increase in venous volume in liver tissue. The pathological features of the model mouse's kidneys induced renal tubular epithelial cell exfoliation (blue arrow), necrotic shedding of renal tubular epithelial cells to form casts (black arrow), and glomerulus pyknosis. There was minimal exfoliation of renal tubular epithelial cells in renal tissue following treatment with HMPDA@NAD^+^@LSA NPs. No pathological changes were observed in the HMPDA@BA/NAD^+^@LSA NPs group, which was the same as the control group. Alveolar wall thickening and neutrophil accumulation were the pathological features of the lungs in the model group (red arrow). However, there was no alveolar wall thickening or neutrophils aggregation after HMPDA@BA/NAD^+^@LSA NPs treatment. In the model group, the pathological characteristics of the spleen were blurred cortical and medulla borders. After treatment with HMPDA@BA/NAD^+^@LSA NPs, no pathological changes were observed in spleen tissue, which was similar to the control group. These pathological results in the major organs provided sufficient support for the structural and functional recovery of the organs following treatment with the nanoprescription.

In the final stage of sepsis, massive cell apoptosis occurs, which eventually accelerates the onset and progression of organ failure. As shown in Figure 6B and Figure S11 (Supporting Information), the positive apoptosis ratios in liver, kidney, and lung tissue of model groups were 52.0%, 51.3%, and 59.2%, respectively. Following treatment with HMPDA@NAD^+^@LSA NPs, the apoptosis ratios of liver, kidney, and lung tissue were 23.1%, 24.5%, and 19.3%, respectively, indicating that the antioxidant effects of PDA and the anti‐inflammatory and energy supply effects of NAD^+^ may inhibit the cell apoptosis process. Surprisingly, the apoptosis rates of liver, kidney, and lung tissue after treatment with HMPDA@BA/NAD^+^@LSA NPs were 15.2%, 13.4%, and 11.4%, respectively, suggesting that chelation of Ca^2+^ can play a synergistic role in effectively preventing massive cell apoptosis and reducing the risk of multiple organ failure.

### 2.4 In Vivo Therapeutic Mechanisms

LPS‐induced intracellular inflammatory cytokine storm and mitochondrial dysfunction are two key contributors to sepsis, and its activation of the NLRP3 inflammasome and mitochondrial apoptosis pathway is the determining factor in cell death. We investigated its anti‐pyroptosis, anti‐inflammatory, and anti‐apoptosis mechanisms from the following two aspects.

#### 2.4.1 Cell Pyroptosis Signaling Pathway

LPS binds to Toll‐like receptor 4 (TLR4) on the cell membrane during sepsis, activating NF‐*κ*B and elevating NLRP3 mRNA transcription levels.^[^ ^27^ ^]^ Oligomerization of the NLRP3 structural protein causes it to bind to PYD of the splitter protein ASC, followed by CARD of ASC binding to CARD on pro‐Casp‐1 to form a complete and active NLRP3 inflammasome, which promotes the self‐cleavage of pro‐Casp‐1, thereby producing the active effector protein Casp‐1.^[^ ^28^ ^]^ Casp‐1 can cleave GSDMD to release N‐GSDMD, which combines with phosphatidylserine and phosphoinositol in the cell membrane, punches holes in the cell membrane, resulting in internal and external imbalance and cell rupture, causing pyroptosis, and releasing contents into the extracellular space to induce inflammation.^[^ ^29^ ^]^ In addition to splitting GSDMD, Casp‐1 can induce the maturation of IL‐1*β* and IL‐18 from immature to an active state.^[^ ^30^ ^]^ Following cell death, IL‐1*β* and IL‐18 are secreted into the extracellular space to recruit inflammatory cells, which then assemble and amplify the inflammatory response.^[^ ^31^ ^]^

**Figure 7:**
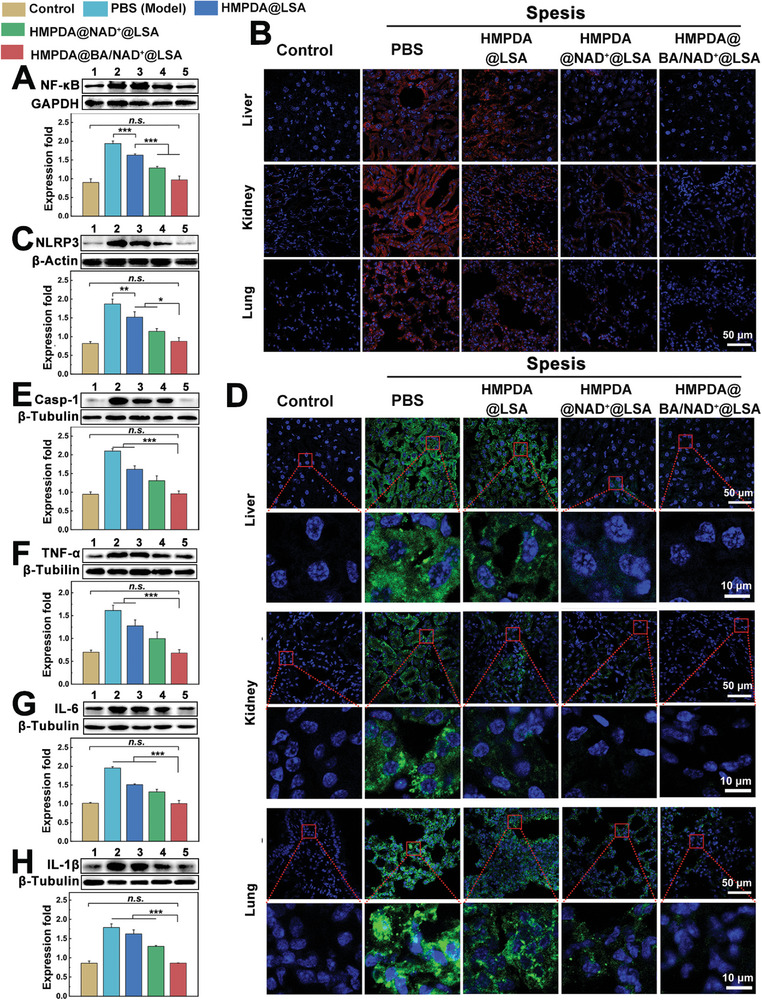
In vivo therapeutic mechanisms. A) Western blotting analysis of NF‐*κ*B expression and semiquantitative results. Data are mean ± SD, *n* = 3. B) Immunofluorescent staining images of NLRP3 in the liver, kidney, and lung (red fluorescence: NLRP3; blue fluorescence: cell nucleus). Data are mean ± SD, *n* = 6. C) Western blotting analysis of NLRP3 expression and semiquantitative results. Data are mean ± SD, *n* = 3. D) Immunofluorescent staining images of the ASC of the liver, kidney, and lung (green fluorescence: the ASC; blue fluorescence: the cell nucleus). Data are mean ± SD, *n* = 6. Western blotting analysis of E) Casp‐1, F) TNF‐*α*, G) IL‐6, and H) IL‐1*β* expression and semiquantitative results. 1–5 represent the control group, the sepsis (PBS) group, the HMPDA@LSA group, the HMPDA@NAD^+^@LSA group, and the HMPDA@BA/NAD^+^@LSA group, respectively. Data are mean ± SD, *n* = 3.

As shown in **Figure** **7**A, NF‐*κ*B expression in the model group was two times that of the control group. After treatment with HMPDA@LSA NPs, the level of NF‐*κ*B decreased by ≈15%, suggesting that ROS clearance alone can only slightly inhibit the NF‐*κ*B expression. NF‐*κ*B in mice treated with HMPDA@NAD^+^@LSA NPs decreased by nearly 37.5%, demonstrating that the added supplement of NAD^+^ significantly inhibited the expression of NF‐*κ*B, as NAD^+^ is not only an energy supplement but also a potent anti‐inflammatory agent. Surprisingly, NF‐*κ*B expression was reduced to healthy levels after HMPDA@BA/NAD^+^@LSA NPs treatment, manifesting that the Ca^2+^ chelation effect provided by BA further inhibited NF‐*κ*B pathway activation. As shown in Figure 7B and Figures S11–S14 (Supporting Information), the expression level of NLRP3 in the liver, kidney, and lung tissue of the model group was 27, 24, and 11 times that of the control group, respectively. The expression of NLRP3 in mice treated with HMPDA@BA/NAD^+^@LSA NPs was not significantly different from that of healthy mice. In addition, NLRP3 expression in liver was specifically assessed by western blotting, and it was shown that the relative NLRP3 expression also recovered to normal level following HMPDA@BA/NAD^+^@LSA NPs treatment (Figure 7C). Considering that the formation of ASC‐speck is essential for the activation of Casp‐1, regulating the formation of ASC‐speck represents a novel strategy for the treatment and prevention of inflammasome‐related diseases. Immunofluorescence observation revealed an abundance of ASC spot‐like proteins (green fluorescence signal) in the liver, kidney, and lung sections of the model group and the HMPDA@LSA group. Few ASC‐speck were identified in the HMPDA@NAD^+^@LSA NPs treatment group, indicating that NAD^+^ effectively suppressed ASC‐speck proteins formation (relative fluorescence intensity of liver, kidney, and lung decreased by 73.1%, 72.1%, and 75.4%, respectively, compared to the model group) (Figure 7D and Figures S15–S18, Supporting Information). Encouragingly, there was almost no detectable ACS‐speck proteins expression in the dual‐drug NPs treatment group (relative fluorescence intensity of liver, kidney, and lung decreased by 92.1%, 86.1%, and 94.4%, respectively, compared with model group), suggesting that chelation of Ca^2+^ by BA could further cascade amplify the inhibition action of HMPDA@NAD^+^@LSA NPs on expression of ASC‐speck proteins.

Casp‐1, the central component of pyroptosis, was expressed twofold as much in the model group compared to the control group, confirming the activation of the pyroptosis pathway in sepsis. Similarly, the content of Casp‐1 in the NAD^+^‐loaded NPs treatment group was decreased by 42.8%, and that in the double‐drug loaded NPs treatment group was decreased by 57.1%, which was consistent with the above‐mentioned protein expression (NF‐*κ*B, NLRP3, and ASC) (Figure 7E), thereby inhibiting the pyroptosis pathway and further inhibiting the subsequent inflammatory response, namely, the downregulation of TNF‐*α* and IL‐6 (Figure 7F,G). Compared with the model group, the expression of TNF‐*α* and IL‐6 after treatment with dual‐drug loaded NPs decreased by 56.3% and 48.6%, respectively. Moreover, the downregulation of Casp‐1 inhibited the expression of IL‐1*β* further downstream. As shown in Figure 7H, the expression of IL‐1*β* in the HMPDA@BA/NAD^+^@LSA NPs treatment group was returned to near normal level, thus inhibiting the subsequent recruitment of inflammatory cells. According to the global expression of the aforementioned pyrocytosis proteins, NAD^+^ was the primary anti‐pyrocytosis component of the nanoagent, while PDA and BA played auxiliary roles, enhancing the anti‐pyrocytosis properties of the nanodrug.

#### 2.4.2 Mitochondrial Apoptosis Signaling Pathway

When cells are stimulated by internal apoptotic factors (ROS, overloaded Ca^2+^, etc.), Bax migrates to the mitochondrial surface, causing irreversible overopening of MPTP, which impedes ATP synthesis, causes massive release of Cyt‐C, and results in massive production of ROS.^[^ ^32^ ^]^ Cyt‐C and apoptotic protease activating factor‐1 (Apaf‐1) combined to form an apoptotic complex, which activated the precursor of Casp‐9, which in turn activated Casp‐3 and Casp‐7, thereby triggering a caspase cascade reaction and inducing cell apoptosis.^[^ ^33^ ^]^

**Figure 8:**
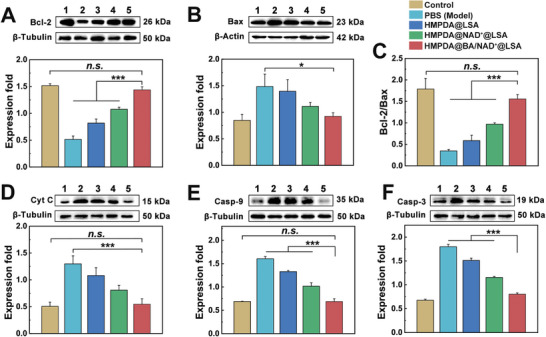
In vivo therapeutic mechanisms. Western blotting analysis of A) Bcl‐2, B) Bax, C) Bcl‐2/Bax, D) Cyt0‐C, E) Casp‐9, and F) Casp‐3 expression, and semiquantitative results. 1–5 represent the control group, the sepsis (PBS) group, the HMPDA@LSA group, the HMPDA@NAD^+^@LSA group, and the HMPDA@BA/NAD^+^@LSA group, respectively. Data are mean ± SD, *n* = 3.

As shown in **Figure** **8**A,B, the expression level of Bcl‐2 (an anti‐apoptotic protein) was significantly decreased in the sepsis model group (about 33% of the expression level of normal mice), whereas the expression level of Bax (a pro‐apoptotic protein) was significantly increased (about two times the expression level of normal mice). Moreover, we compared the Bcl‐2/Bax ratio, which determined whether cells survived apoptotic stimulation or perished. The stronger the anti‐apoptotic capability of cells was indicated by a higher ratio. As shown in Figure 8C, after treatment with HMPDA@BA/NAD^+^@LSA NPs, the value of Bcl‐2/Bax in the sepsis mice was significantly increased, about four times that of the model group, indicating that the anti‐apoptotic ability of the cells was significantly enhanced after treatment. The Cyt‐C expression level in the model group was about 2.5 times that of the control group, whereas the Cyt‐C expression level in the NAD^+^‐loaded NPs treatment group or the NAD^+^ and BA‐AM co‐loaded NPs treatment group was reduced by 42.9% and 64.3%, respectively, when compared to the model group (Figure 8D). As shown in Figure 8E, the content of Casp‐9 in the sepsis model group was 2.4 times that of the control group, whereas the expression of Casp‐9 in the HMPDA@BA/NAD^+^@LSA NPs group did not differ significantly from that of the control group. The expression of Casp‐3 at the end of the mitochondrial apoptotic pathway in each treatment group was consistent with that of Casp‐9 (Figure 8F). Above results demonstrated that NPs inhibited the release of upstream apoptotic factors Bax and Cyt‐C by depleting intracellular ROS, restoring cell vitality, and chelating intracellular Ca^2+^, further downregulating the expression of downstream apoptotic proteins Casp‐9 and Casp‐3, and ultimately achieving the purpose of blocking the mitochondrial apoptosis pathway.

**Figure 9:**
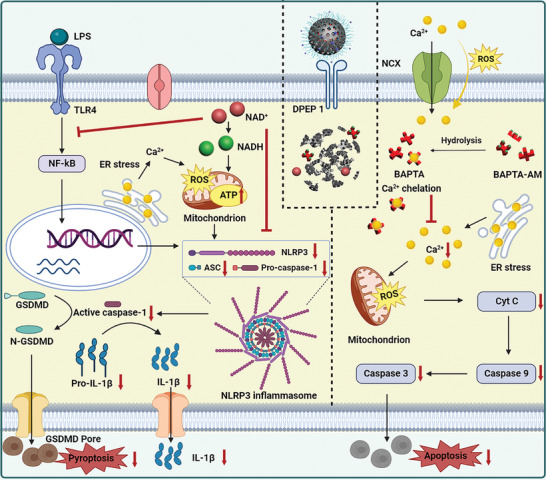
Schematic illustration of cellular regulatory mechanism of HMPDA@BA/NAD^+^@LSA NPs.

In conclusion, we have fully validated the potential molecular mechanism of HMPDA@BA/NAD^+^@LSA NPs to alleviate multiple organ damage in sepsis (**Figure** **9**), concluding that the above NPs exert excellent anti‐inflammatory, antioxidant, and anti‐apoptotic effects in vivo by inhibiting the activation of pyroptosis pathways, inflammatory pathways, and mitochondrial apoptosis pathways.

### 2.5 In Vivo Biosafety Evaluation

Calcium antagonists are commonly used to treat hypertension in clinical practice, but an overdose can cause hypotension and even cardiac arrest.^[^ ^34^ ^]^ Therefore, we evaluated the effects of BA‐AM‐containing NPs on the blood pressure and heart rate of mice. HMPDA@BA/NAD^+^@LSA was injected into healthy mice at doses of 100, 200, and 300 µg kg^−1^ for BA‐AM. As shown in Figure S20 (Supporting Information), even at the highest dose of BA‐AM (300 µg kg^−1^), the effects on blood pressure (mean arterial pressure, MAP), and heart rate in mice were not significantly different from those of normal mice. The results showed that HMPDA@BA/NAD^+^@LSA NPs with a single dose of BA‐AM concentration of 200 µg kg^−1^ had no cardiovascular adverse effects in a mouse model of sepsis, a requirement for future clinical transformation.

## 3 Conclusion

In this study, we developed a drug delivery system that targets the pathogenesis of sepsis and addressed the following key issues: i) efficient co‐loading of hydrophilic and hydrophobic drugs; ii) active targeting: precise delivery of nanomedicine to the injury site; iii) acid‐responsive drug release: depolymerization of HMPDA under slightly acidic inflammatory conditions; iv) and precise therapy: suppress sepsis multi‐organ failure from the perspective of inhibiting pyroptosis and rescuing mitochondrial dysfunction, thereby improving the efficacy of sepsis treatment.

Why does this nanoagent inhibit inflammation, pyroptosis and apoptosis so effectively as sepsis progression? i) NAD^+^, an effective anti‐inflammatory agent, prevented the formation of the NLRP3 inflammasome, thus inhibiting the expression of the downstream effector protein Casp‐1 and significantly interfering with the pyroptosis process. Besides, the downregulation of Casp‐1 further prevented the subsequent outbreak of an inflammatory storm. Obviously, HMPDA and BA‐AM also enhance the anti‐inflammatory and anti‐pyroptosis effects of NAD^+^ through a synergistic effect. ii) The depletion of ROS by PDA, chelation of Ca^2+^ by BA‐AM, and replenishment of NAD^+^ on the mitochondrial energy pool rapidly restored mitochondrial function from the source, cut off the conduction of key apoptotic signals (Bax‐Cyt‐C‐Casp‐3 signal axis), and finally successfully inhibited the mitochondrial apoptosis pathway.

In our experiment, a low dose of nanopreparation (HMPDA: 320 µg kg^−1^; BA‐AM: 200 µg kg^−1^; NAD^+^: 320 µg kg^−1^) effectively inhibited both the pyroptosis pathway and the mitochondrial apoptosis pathway, restoring organ function rapidly to its normal level. In addition, there were no acute cardiovascular side effects observed. We are confident that this formulation has great clinical value in the treatment of sepsis‐induced multiple organ failure.

## 4 Experimental Section

### Material

Dopamine hydrochloride, Pluronic F‐127, BAPTA‐AM, and nicotinamide adenine dinucleotide (NAD^+^) were purchased from Aladdin Reagents (Shanghai, China). Tris (hydroxymethyl) aminomethane (TRIS) and 1,3,5‐trimethylbenzene (TMB) were purchased from Sigma‐Aldrich (USA). NH_2_‐PEG‐Mal (Mw: 2000 Da) was purchased from Ruixi Biotech Co., Ltd. CLSALTPSPSWLKYKAL (Cys‐LSALT) was purchased from Nanjing Peptide Biotech Ltd., Total Antioxidant Capacity Assay Kit, Calcein‐AM/PI Cell Viability/Cytotoxicity Assay Kit, CCK‐8, Fluo‐4 AM, *H&E* Staining Kit, One‐Step TUNEL Cell Apoptosis Detection Kit, Dihydroethidium fluorescent probe, Mitochondrial membrane potential assay kit, ATP Assay Kit, and NAD^+^/NADH Assay Kit with WST‐8 were provided by Beyotime Institute of Biotechnology. Urea nitrogen kit, Creatinine Assay kit, MDA assay kit, and SOD assay kit were purchased from Nanjing Jiancheng Bioengineering Institute. AKP/ALP activity detection kit, the Micro GOT/AST assay kit, the Micro GPT/ALT assay kit, and the DCFH‐DA reactive oxygen fluorescence probe were purchased from Beijing Solarbio Science & Technology Co., Ltd. Anti‐NLRP3 antibody, anti‐Casp‐1 antibody, anti‐IL‐1*β* antibody, anti‐Cyt‐C antibody, anti‐Casp‐9 antibody, anti‐Casp‐3 antibody, anti‐Bax antibody, anti‐TNF‐ɑ antibody, anti‐NF‐*κ*B antibody, and anti‐*β*‐Tubulin Ab were provided by Wanlei Biotechnology Co., Ltd. Anti‐Bcl‐2 antibody, goat anti‐rabbit IgG‐HRP, and goat anti‐mouse IgG‐HRP were provided by Absin biotech. Co., Ltd. Anti‐GAPDH antibody and anti‐*β*‐Actin antibody were provided by Servicebio. Co., Ltd. Anti‐IL‐6 antibody was provided by Boster Biological Technology Co., Ltd. All reagents were of analytical grade and used without any purification.

### Preparation and Characterization of HMPDA@BA/NAD^+^@LSA NPs

*HMPDA Synthesis*: A small HMPDA was synthesized using a modified version of the soft template method, as previously described.^[^ ^35^ ^]^ Under stirring, 0.2 g F127 and 0.2 g TMB were added to a mixture solution (50 mL, *V*_ethanol_:*V*_deionized water_ = 1:1). Then, 45 mg of Tris dissolved in 10 mL of water was added to the mixture, followed by the addition of 60 mg of dopamine hydrochloride for 24 h. The mixture was centrifuged at 13 000 rpm min^−1^ and washed with a mixed solution (*V*_ethanol_:*V*_acetone_ = 2:1) by ultrasonic agitation for 30 min per time, three times. The final product was suspended in water for further use.

*BA‐AM Loading in HMPDA*: 4 mg BA‐AM was dissolved in 2 mL DMSO and added to 2 mL HMPDA aqueous dispersion (1 mg mL^−1^) under ultrasound. The mixture was then stirred at room temperature for 1 h, centrifuged, and washed to obtain HMPDA@BA NPs. The content of BA‐AM in the supernatant (free BA‐AM) and HMPDA@BA NPs sediment (loaded BA‐AM) were determined by HPLC (Agilent 1200, USA), respectively. The HPLC conditions were as follows: column, C18 (250 mm × 4.6 mm, 5 µm); injection volume, 5 µL; column temperature, 40 °C; mobile phase (containing 0.1% trifluoroacetic acid): acetonitrile:deionized water = 3:2; detection wavelength: 254 nm. The drug encapsulation efficiency (EE) and LC of BA‐AM in HMPDA@BA NPs were calculated according to the following equation

(2) $$\mathrm{EE}\;\left(\%\right)=\frac{\left[\mathrm{Weight}\;\mathrm{of}\;\mathrm{encapsulated}\;\mathrm{drug}\right]}{\left[\mathrm{Total}\;\mathrm{weight}\;\mathrm{of}\;\mathrm{drug}\right]}\times 100\%$$

(3) $$\mathrm{LC}\;\left(\%\right)=\frac{\mathrm{Weight}\;\mathrm{of}\;\mathrm{encapsulated}\;\mathrm{drug}}{\mathrm{Total}\;\mathrm{weight}\;\mathrm{of}\;\mathrm{nanoparticles}}\times 100\%$$

*NAD^+^ Loading in HMPDA*: After dissolving 10 mg of NAD^+^ in 1 mL of ultrapure water, it was added to 1 mL of HMPDA dispersed solution (2 mg mL^−1^). After 1 h of reaction at room temperature, NPs containing NAD^+^ (HMPDA@NAD^+^) were centrifuged (13 000 rpm, 30 min), washed in ultrapure water, and centrifuged again for 30 min. The content of NAD^+^ in the supernatant (free NAD^+^) and HMPDA@NAD^+^ NPs sediment (loaded NAD^+^) was determined by HPLC (Agilent 1200, USA), respectively. HPLC conditions were as follows: column, C18 (250 mm × 4.6 mm, 5 µm); injection volume, 5 µL; column temperature, 40 °C; mobile phase A, sodium dihydrogen phosphate; mobile phase B, methanol; detection wavelength: 260 nm. The drug EE and LC of NAD^+^ in HMPDA@NAD^+^ NPs were calculated according to Equations (2) and (3).

*BA‐AM and NAD^+^ Co‐Loading in HMPDA*: 8 mg of HMPDA@BA was dispersed in 2.5 mL of ultrapure water, followed by the addition of 2.5 mL of a 10 mg mL^−1^ NAD^+^ aqueous solution. After 1 h of stirring at room temperature, the HMPDA@BA/NAD^+^ NPs were collected by centrifugation and subsequently dispersed in water for further use. After the initial centrifugation, the supernatant was collected. The content of NAD^+^ in the supernatant (free NAD^+^) was determined by HPLC (Agilent 1200, USA), respectively. The drug EE of NAD^+^ in HMPDA@BA/NAD^+^ NPs was calculated according to Equation (2).

*Surface Modification of the DPEP‐1 Targeting Peptide*: HMPDA@BA/NAD^+^ modified by the targeting peptide Cys‐LSALT (Cys‐LSA) was prepared via a two‐step reaction. PEG was initially used to covalently modify the surface of synthesized HMPDA@BA/NAD^+^. Specifically, 2 mg HMPDA@BA/NAD^+^ was dispersed in 1 mL of TRIS solution (10 × 10^−3^ m, pH 8.5), followed by the addition of 1 mL of NH_2_‐PEG‐Mal TRIS solution (10 mg mL^−1^) and 24 h of stirring at room temperature. The PEGylated HMPDA@BA/NAD^+^ NPs were obtained by centrifugation and washing. To determine whether NH_2_‐PEG‐Mal was successfully grafted, the UV absorption (215 nm) of NH_2_‐PEG‐Mal (5 mg mL^−1^) dissolved in TRIS solution and the supernatant after grafting were measured, respectively. Second, PEGylation HMPDA@BA/NAD^+^ and 0.2 mg LSA were dispersed in 2 mL ultrapure water, magnetically stirred at room temperature for 2 h, centrifuged, and washed three times to obtain NPs modified by LSA (HMPDA@BA/NAD^+^@LSA). To determine whether Cys‐LSA successfully grafted, the UV absorption (197 nm) of Cys‐LSA (0.1 mg mL^−1^) dissolved in water and the supernatant after grafting were measured, respectively.

*Synthesis of the DiR‐Loaded NPs*: In the process of loading BA‐AM, 0.5 mg of DiR and BA‐AM were put into it simultaneously, and the rest remained unchanged according to the step “BA‐AM and NAD^+^ Co‐Loading in HMPDA.” Then, the above NPs were PEGylated according to the step “Surface Modification of the DPEP‐1 Targeting Peptide” to obtain HMPDA@DiR/BA/NAD^+^ NPs. In addition, the steps of “Surface Modification of the DPEP‐1 Targeting Peptide” were followed exactly to obtain HMPDA@DiR/BA/NAD^+^@LSA NPs.

*Characterization of NPs: Particle Size, ζ‐Potential, and Surface Morphology Analysis of NPs*: The size distribution and surface charge density of NPs were determined by a Malvern Zetasizer Nano Instrument (ZEN3600, Malvern, UK) The size distribution and morphology of NPs were further measured by transmission electron microscopy (HT7700, Hitachi, Japan).

*Specific Surface Area and the Pore Size Analysis of NPs*: The specific surface area and pore size distributions of NPs were measured using a fully automated BET surface (volume) analyzer (NOVA2200e, Quantachrome, USA).

*pH Responsiveness In Vitro*: DLS measurements were performed for 1 h at 37 °C after exposing HMPDA NPs to the following buffer solutions: PBS (pH 7.4), PBS (pH 6.5), and PBS (pH 5.5). After 6 h of incubation, the acid responsiveness of the HMPDA NPs was further evaluated by observing the changes in the “Tyndall Effect” generated by laser beam irradiation.

*In Vitro Drug Release*: HMPDA@BA/NAD^+^@LSA NPs (30 mg) were distributed in 30 mL of PBS containing 0.5% Tween 80 and uniformly placed in three dialysis bags. Then, these dialysis bags were dialyzed in PBS buffer solutions (100 mL) with varied pH values (7.4, 6.5, and 5.5) at 37 °C. At predetermined time intervals (0, 1, 3, 6, 9, 12, 24, 36, and 48 h), 2 mL of buffer solution was withdrawn and replaced with 2 mL of fresh buffer solution. The extracted buffer solution (divided into two parts) was freeze‐dried and resuspended with ultrapure water and DMSO to determine the concentrations of NAD^+^ and BA‐AM by HPLC, respectively. All results for drug release were obtained from three independent experiments.

*ROS Consumption Capacity In Vitro*: ABTS can be used as a chromogenic agent to determine the total antioxidant capacity of various substances. The antioxidant capacity of a 0.1 mg mL^−1^ HMPDA PBS dispersion was measured at different pH values according to the manufacturer's protocol.

*Storage Stability*: To determine the storage stability of NPs, the particle size and PDI changes of NPs measured by DLS were monitored for one week at room temperature (25 °C).

### Cell Study

*Cell Culture*: Human Kidney‐2 cells (HK‐2) and Alpha Mouse Liver 12 cells (AML‐12) were cultured with DMEM supplemented with 10% (v/v) FBS and penicillin/streptomycin (100 IU mL^−1^). All cells were cultured in a cell culture at 37 °C and 5% CO_2_.

*In Vitro Biocompatibility: Cytotoxicity Assays*: CCK‐8 assays were utilized to determine the cytotoxicity of HMPDA@LSA and HMPDA@BA/NAD^+^@LSA NPs. Simply, HK‐2 cells were seeded at 2 × 10^4^ per well and cultured in 96‐well plates for 12 h. HMPDA@LSA and HMPDA@BA/NAD^+^@LSA NPs were then diluted to final HMPDA concentrations of 0, 1, 2, 5, 10, 20, 50, and 100 µg mL^−1^ in cell medium, respectively, and incubated for 24 h. The CCK‐8 kit was subsequently used to assess the cytotoxicity of HMPDA@LSA and HMPDA@BA/NAD^+^@LSA NPs toward HK‐2 cell lines. AML‐12 cells were substituted for HK‐2 cells, and the cytotoxicity of NPs on AML‐12 cell lines was determined without changing other steps.

*Hemolysis Test*: Red blood cells (RBC) were diluted with PBS to a concentration of 4%. At 37 °C for 1 h, 0.5 mL of RBC dilution was incubated with a series of HMPDA@BA/NAD^+^@LSA NP concentrations (0.5 mL) ranging from 12.5 to 500 µg·mL^−1^. As positive controls (100% hemolysis) and negative controls (0% hemolysis), respectively, 0.1% Triton X‐100 and PBS were used. After 10 min of centrifugation at 3000 rpm, the absorbance of the supernatant at 576 nm was measured. The calculation formula for the hemolysis rate is as follows

(4) $$\begin{array}{c} \mathrm{Hemolysis}\;\left(\%\right)=\frac{\left(A576\;\mathrm{of}\;\mathrm{sample}-A576\;\mathrm{of}\;\mathrm{negative}\right)}{\left(A576\;\mathrm{of}\;\mathrm{positive}-A576\;\mathrm{of}\;\mathrm{negative}\right)}\times 100\% \end{array}$$

*Protective Effect against Liver Cell Injury In Vitro: Establishment of a Model of Liver Cell Injury*: AML‐12 cells were seeded at a density of 1 × 10^6^ per well in six‐well plates and cultured for 12 h. Cells were pretreated with H_2_O_2_ (200 × 10^−6^ m) for 2 h. Then the H_2_O_2_‐stimulated AML‐12 cells were incubated with different groups for 12 h (Control group, PBS group, HMPDA@LSA group (HMPDA: 320 ng mL^−1^), free NAD^+^ group (320 ng mL^−1^), free BA‐AM group (200 ng mL^−1^), HMPDA@NAD^+^@LSA group (NAD^+^: 320 ng mL^−1^), HMPDA@BA@LSA group (BA‐AM: 200 ng mL^−1^), HMPDA@BA/NAD^+^@LSA group (BA‐AM: 200 ng mL^−1^, NAD^+^: 320 ng mL^−1^).

### Determination of Mitochondrial Membrane Potential

The MMP probe JC‐1 was employed to validate the ability of NPs to restore membrane potential through semiquantitative analysis. H_2_O_2_‐stimulated cells were treated with the aforementioned formulations (Section “Establishment of a model of liver cell injury”) for 12 h to simulate acute liver injury. After discarding the medium and adding JC‐1 for 30 min of incubation, cells were washed three times with PBS and observed under an inverted fluorescent microscope (ICX41, SOPTOP, China). The relative fluorescence intensity of MMP in six randomly selected fields of vision was semiquantified by ImageJ software (National Institutes of Health, USA).

### Assessment of Intracellular NAD^+^/NADH Levels

The NAD^+^/NADH Assay Kit with WST‐8 was used to detect the amount of NAD^+^ and NADH in cells by the colorimetric method. Simulating acute liver injury, H_2_O_2_‐stimulated cells were treated for 12 h with the aforementioned formulations (Section “Establishment of a model of liver cell injury”), and the amount of NAD^+^ and NADH in the cells was measured according to the manufacturer's protocol.

### Assessment of Intracellular ATP levels

Intracellular ATP levels were measured with an ATP assay kit. Simulating acute liver injury, H_2_O_2_‐stimulated cells were treated with the aforementioned formulations for 12 h (Section “Establishment of a model of liver cell injury”), and the amount of ATP in the cells was measured according to the manufacturer's protocol.

### Cytoprotective Effects

In 96‐well plates, AML‐12 cells were seeded at a density of 2 × 10^4^ per well and cultured for 12 h. After 12 h of treatment with the aforementioned formulations (Section “Establishment of a model of liver cell injury”), the media were aspirated, the cells were washed three times with PBS, and CCK‐8 solution was added. After incubating the 96‐well plate for 1 h in an incubator, the absorbance at 450 nm was measured using a microplate reader.

*Protective Effect against Renal Cell Injury In Vitro: Establishment of a Model of Renal Cell Injury*: HK‐2 cells were seeded at a density of 1 × 10^6^ per well in six‐well plates and cultured for 12 h. Cells were pretreated with H_2_O_2_ (200 × 10^−6^ m) for 2 h. Then the H_2_O_2_‐stimulated HK‐2 cells were incubated with different groups for 12 h (control group, PBS group, HMPDA@LSA group (HMPDA: 320 ng mL^−1^), free NAD^+^ group (320 ng mL^−1^), free BA‐AM group (200 ng mL^−1^), HMPDA@NAD^+^@LSA group (NAD^+^: 320 ng mL^−1^), HMPDA@BA@LSA group (BA‐AM: 200 ng mL^−1^), HMPDA@BA/NAD^+^@LSA group (BA‐AM: 200 ng mL^−1^, NAD^+^: 320 ng mL^−1^).

*Assessment of Intracellular Ca^2+^ Levels*: To evaluate the ability of NPs to chelate intracellular Ca^2+^, H_2_O_2_‐stimulated cells modeling acute kidney injury were treated with the formula described in section “Establishment of a model of renal cell injury” for 12 h, then loaded with Fluo‐4 AM, collected, and suspended in PBS. Intracellular relative Ca^2+^ levels were analyzed by flow cytometry (Accuri C6, BD, USA).

*In Vitro Antioxidant Performance*: ROS levels in cells were determined by the ROS probe (DCFH‐DA) to validate the ability of NPs to scavenge ROS. To simulate acute kidney injury, H_2_O_2_‐stimulated cells were treated with the formulations described in section “Establishment of a model of renal cell injury” for 12 h, then loaded with DCFH‐DA, stained with DAPI, and washed with PBS three times. The cells were observed under an inverted fluorescence microscope (ICX41, SOPTOP, China). Fluorescence images were semiquantified with the ImageJ software or the relative fluorescence intensity of ROS in six randomly selected visual fields.

*Cytoprotective Effects*: In 96‐well plates, HK‐2 cells were seeded at a density of 2 × 10^4^ per well and cultured for 12 h. H_2_O_2_‐stimulated cells were treated for 12 h with the aforementioned formulations (section “Establishment of a model of renal cell injury”), and media were aspirated, washed three times with PBS, and a CCK‐8 solution was added. After placing the 96‐well plate in an incubator for 2 h, the absorbance at 450 nm was measured using a microplate reader. To investigate cellular viability, the Calcein‐AM/PI Kit (2 µmol L^−1^ PI and 1 µmol L^−1^ Calcein‐AM probe) was utilized. Six random fields were selected, and photographed under an inverted fluorescence microscope (ICX41, SOPTOP, China), and quantified using ImageJ software (National Institutes of Health, USA). The cell viability rate was calculated by dividing the number of live cells by the total number of cells per field.

### Animal Studies

The ICR mice (6–7 weeks old, 30–35 g, male) were supplied by the Qingdao Qinda Biotechnology Co., Ltd. (Qingdao, China). All rat experiments were conducted in compliance with the Use and Care of Experiments. The animal experiments were approved by the Institutional Animal Care and Use Committee of the Ocean University of China.

*LPS‐Induced Sepsis Animal Model*: The LPS‐induced sepsis mouse model was established as previously described.^[^ ^36^ ^]^ Mice were randomly grouped according to body weight and then given LPS of 15 mg kg^−1^ (Sigma‐Aldrich) intravenously to induce sepsis.

*In Vivo Biodistribution*: The HMPDA@BA/NAD^+^@LSA NPs or HMPDA@BA/NAD^+^ NPs loaded with a DiR NIR probe (ex: 748 nm, em: 780 nm) were prepared. The mice were divided into three groups: 1) healthy mice + HMPDA@DiR/BA/NAD^+^@LSA NPs; 2) LPS‐induced sepsis mice + HMPDA@DiR/BA/NAD^+^ NPs; and 3) LPS‐induced sepsis mice + HMPDA@DiR/BA/NAD^+^@LSA NPs. The mice were sacrificed at 3 and 6 h following NPs administration. Major organs were collected for ex vivo imaging with a multifunctional fluorescence imaging system (Fusion FX7, Vilber Lourmat, USA).

*Experimental Group*: Mice were randomly assigned to one of six groups: 1) Control group; 2) Model (PBS) group; 3) sepsis model + HMPDA@LSA NPs (HMPDA: 320 µg kg^−1^); 4) sepsis model + HMPDA@BA@LSA NPs (BA‐AM: 200 µg kg^−1^); 5) sepsis model + HMPDA@NAD^+^@LSA NPs (NAD^+^: 320 µg kg^−1^); 6) sepsis model + HMPDA@BA/NAD^+^@LSA NPs (BA‐AM: 200 µg kg^−1^; NAD^+^: 320 µg kg^−1^). The retro‐orbital venous plexus was punctured to collect blood samples prior to modeling (0 h) and 6 and 12 h after the initial injection. The mice were sacrificed after 24 h, and the kidney, liver, and lung were collected and cleaned with PBS. Part of the organs were immediately weighed with their wet weight and dried in an oven at 60 °C for 24 h before being weighed with their dry weight. The wet/dry ratio of organs was calculated using the following equation

(5) $$\mathrm{Wet}/\mathrm{Dry}\;\mathrm{ratio}=\frac{\mathrm{Wet}\;\mathrm{weight}\;\left(\mathrm{mg}\right)}{\mathrm{Dry}\;\mathrm{weight}\;\left(\mathrm{mg}\right)}\times 100\%$$

The remaining liver, kidney, and lung were divided into three parts: 1) frozen for WB and tissue homogenate; 2) embedded with OTC glue and frozen in liquid nitrogen for frozen section; and 3) immersed in 4% paraformaldehyde for H&E sectioning. In addition, the spleen was collected and immersed in paraformaldehyde for the H&E section.

*Detection of Renal Function*: BUN and GRE levels in serum collected from mice 6 and 12 h after treatment with different formulations were measured according to the manufacturer's protocol to assess the recovery of renal function.

*Detection of Liver Function*: AST and ALT levels in serum collected from mice at 6 and 12 h after treatment with different formulations and AKP levels in different treatment groups’ liver homogenates were measured according to the manufacturer's protocol to assess the recovery of liver function.

*In Vivo Antioxidant Determination*: Fresh liver, kidney, and lung tissue were embedded in OTC, sectioned with a cryostat microtome (Leica, CM1860, Germany), and stained with DHE and DAPI. The images were observed and photographed using a fluorescence microscope, and the average fluorescence intensity of six randomly selected fields of view was calculated using ImageJ software (National Institutes of Health, USA). The expression levels of antioxidant damage indicators (SOD and MDA) in each treatment group were determined using the liver tissue collected for tissue homogenate in the section “Experimental group.”

*Detection of Inflammasome‐Associated Protein Expression*: Liver, renal, and lung tissues in OTC were sectioned with a cryostatic microtome (5 µm thick). The sections were fixed and sealed, incubated with NLRP3 antibody (37 °C, 1 h), followed by a Cy3‐labeled secondary antibody (37 °C, 1 h), and then stained with DAPI. After staining, these sections were washed five times with PBS, observed and photographed with a confocal laser microscope (NikonA1^+^, Japan), and semiquantitative fluorescence intensity analysis of NLRP3 was performed in six randomly selected fields using ImageJ software (National Institutes of Health, USA). The immunofluorescence steps of ASC remained unchanged except for the substitution of an ACS antibody for the primary antibody and a FITC‐labeled antibody for the secondary antibody.

*Histological Analysis*: Liver, spleen, kidney, and lung tissues were fixed, dehydrated, embedded, sectioned (5 µm thick), stained with H&E, and then captured stained section images using the Vectra Automated Quantitative Pathology Imaging System (Launches Vectra 3, PerkinElmer, USA). At least six visual fields were randomly selected for evaluation.

*Evaluation of Anti‐Apoptosis In Vivo*: OTC‐embedded tissue was sliced using a cryostat microtome, fixed, incubated with protease K at room temperature for 10 min, and then incubated with TdT enzyme solution at 37 °C for 2 h. The slides were stained with DAPI and washed with PBS three times. Utilizing fluorescence microscopy, the percentage of TUNEL‐positive cells was calculated. The number of TUNEL‐positive cells per field and the total number of cells per field were quantified using the ImageJ software.

*Western Blotting Assay*: Protein was extracted from liver homogenates using a protease inhibitor‐containing RIPA buffer, and then quantified by the BCA kit. On a 12/15% SDS‐PAGE gel, protein electrophoresis was performed, and then the protein was transferred to a PVDF membrane (Merck Millipore, USA). The membranes were blocked in 5% skim milk before being incubated overnight at 4 °C with primary anti‐NF‐*κ*B antibody, anti‐NLRP3, anti‐Casp‐1, anti‐IL‐1*β*, anti‐TNF‐*α*, IL‐6, anti‐Bax, anti‐Bcl‐2, anti‐Cyt‐C, anti‐Casp‐9, or anti‐Casp‐3. The membranes were washed and incubated with a secondary antibody (conjugation with HRP) for 1 h at 37 °C. Protein signals were detected using a multi‐chemiluminescence gel imaging system (5200Multi, Tanon, China) and quantified with ImageJ software. The quantitative results of all western blotting analyses were obtained from three independent experiments.

*Monitoring of Cardiovascular Effects*: To assess the effects of this formulation on the cardiovascular system, healthy mice (ICR mouse, 7–8 weeks old, male, 30–35 g) were injected intravenously with HMPDA@BA /NAD^+^@LSA NPs at dosages of 100, 200, and 300 µg kg^−1^ for BA‐AM, respectively. Blood pressure and heart rate were monitored at 0, 1, 3, and 6 h after injection using a noninvasive arterial sphygmomanometer (ZS‐Z, Dichuang, China).

### Statistical Analyses

The data were analyzed and presented as means ± SD. The statistical significance of different groups was determined using One‐way ANOVA with multiple comparisons. *p* < 0.05 were used as the statistically significant difference threshold (*p < 0.05, **p < 0.01, ***p < 0.001).

## Conflict of Interest

The authors declare no conflict of interest.

## Author Contributions

J.Y. and J.Z. contributed equally to this work. J.Y. wrote the manuscript; J.Y., M.P., Y.W., H.L., and X.S. conducted the experiments; P.C., X.Y., L.Y., and Z.H. conceived this project. J.Y. analyzed the data; all authors cowrote the paper with full discussion.

## Supporting information

Supporting Information

## Data Availability

The data that support the findings of this study are available from the corresponding author upon reasonable request.
