# Supplementary material for: Rapidly Inhibiting the Inflammatory Cytokine Storms and Restoring Cellular Homeostasis to Alleviate Sepsis by Blocking Pyroptosis and Mitochondrial Apoptosis Pathways
Source: Adv Sci (Weinh). 2023 Mar 17;10(14):2207448. doi: 10.1002/advs.202207448 (PMC10190643; doi:10.1002/advs.202207448)
Supplement: Supplementary file 1 — Supporting Information [file ADVS-10-2207448-s001.pdf]

ADVANCED SCIENCE
Open Access

Supporting Information

for *Adv. Sci.*, DOI 10.1002/advs.202207448

Rapidly Inhibiting the Inflammatory Cytokine Storms and Restoring Cellular Homeostasis to Alleviate Sepsis by Blocking Pyroptosis and Mitochondrial Apoptosis Pathways

Jiahui Yan, Jingwen Zhang, Yanan Wang, Hong Liu, Xueping Sun, Aixin Li, Pengfei Cui, Liangmin Yu, Xuefeng Yan and Zhiyu He**

Rapidly inhibiting the inflammatory cytokine storms and restoring cellular homeostasis to alleviate sepsis by blocking pyroptosis and mitochondrial apoptosis pathways

Jiahui Yan[a,b‡], Jingwen Zhang[a,b‡], Yanan Wang[a,b], Hong Liu[a,b], Xueping Sun[a,b], Aixin Li[a,b], Pengfei Cui[c], Liangmin Yu[a,b]*, Xuefeng Yan[a,b], and Zhiyu He[a,b]*

[a] Frontiers Science Center for Deep Ocean Multispheres and Earth System, and Key Laboratory of Marine Chemistry Theory and Technology, Ministry of Education, Ocean University of China, Qingdao, China

[b] College of Chemistry and Chemical Engineering, Ocean University of China, Qingdao, China

[c] College of Marine Life Sciences, Ocean University of China, Qingdao, China

E-mail: yuyan@ouc.edu.cn (**L. Yu**); hezhiyu@ouc.edu.cn (**Z. He**).

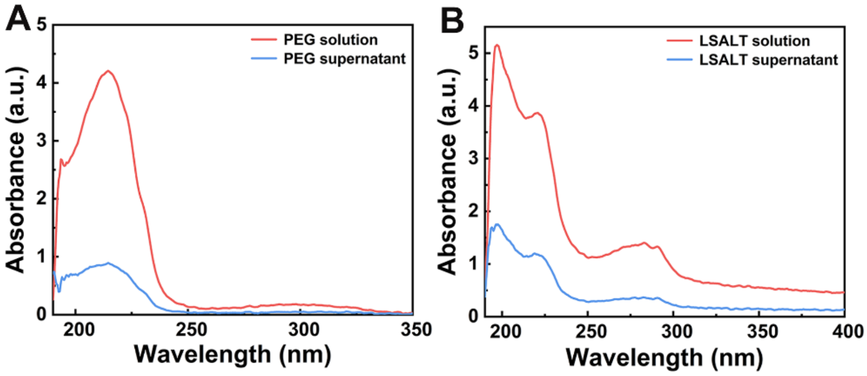

Figure S1: UV-Vis spectra of free a) NH_2-PEG- Mal (PEG) and b) Cys-LSALT (LSA) in the centrifuged supernatant after grafting.

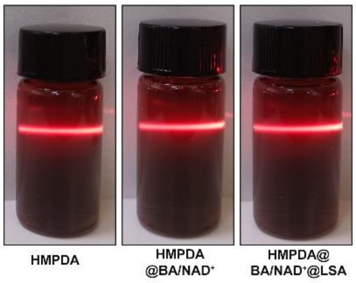

Figure S2: Dundar phenomenon diagram of HMPDA NPs, HMPDA@BA/NAD$^+$ NPs, and HMPDA@BA/NAD$^+$@LSA NPs.

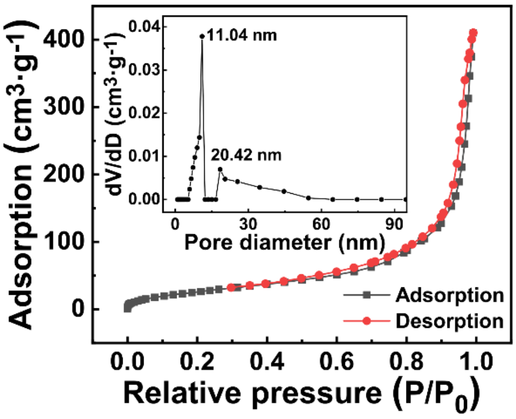

Figure S3: N_2 adsorption-desorption isotherm and pore-size distribution of HMPDA NPs.

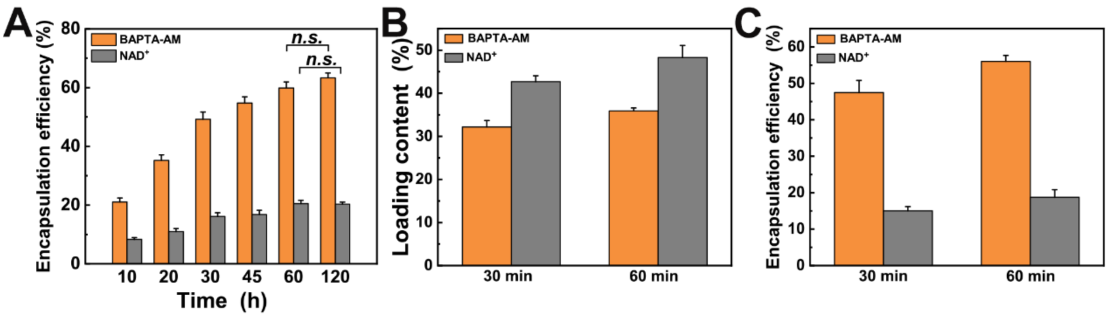

Figure S4: A) Influence of incubating time on encapsulation efficiency (calculated by determination of free drug content) of NAD^+ and BA-AM in HMPDA NPs. B) Loading content and C) encapsulation efficiency of BA-AM and NAD^+ in HMPDA NPs at different incubation times (calculated by determination of the drug content loaded on NPs).

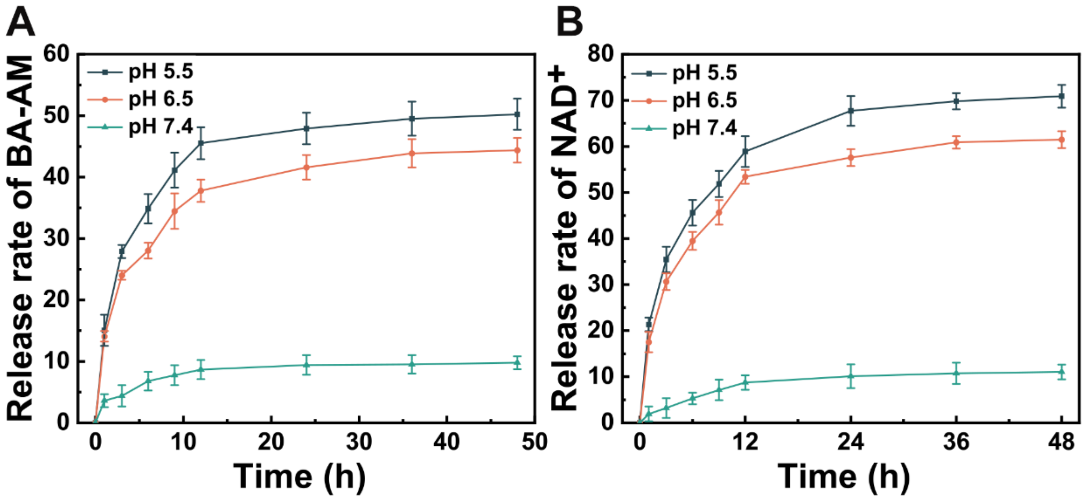

Figure S5: The drug (BA-AM and NAD^+) release curves of HMPDA@BA/NAD^+@LSA NPs incubated in simulated medium (PBS with different pH).

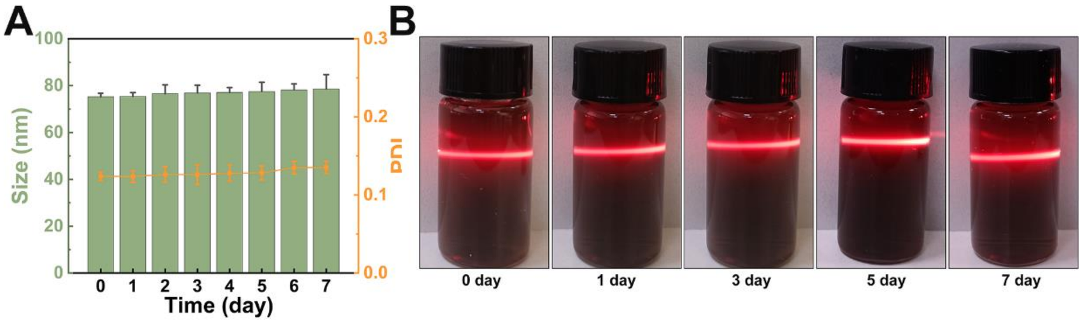

Figure S6: (A) Storage stability and (B) Light path diagram of the "Tyndall effect" of HMPDA@BA/NAD$^+$@LSA NPs in PBS (pH = 7.4, 10 mM) for 1 week at room temperature.

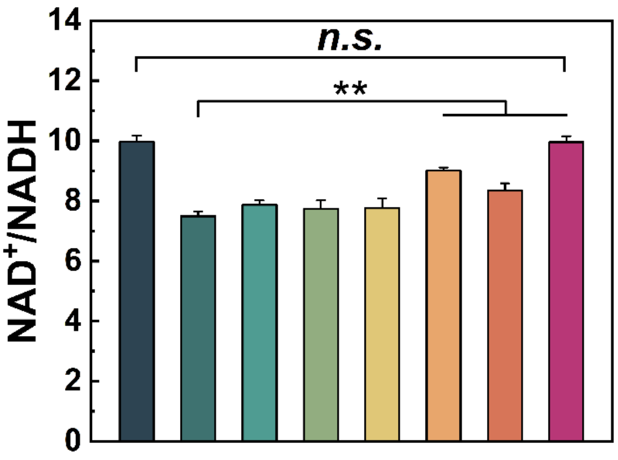

Figure S7: NAD$^+$/NADH level in H_2O_2-stimulated AML-12 cells with different treatments.

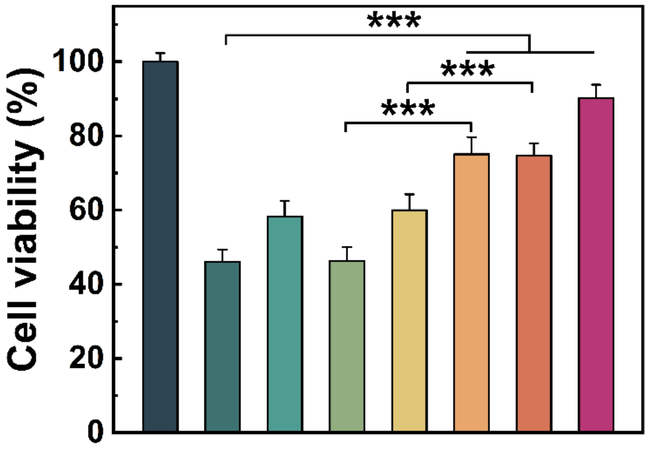

Figure S8: Cell viability of H_2O_2-induced acute injured AML-12 cells after 12 h treatment.

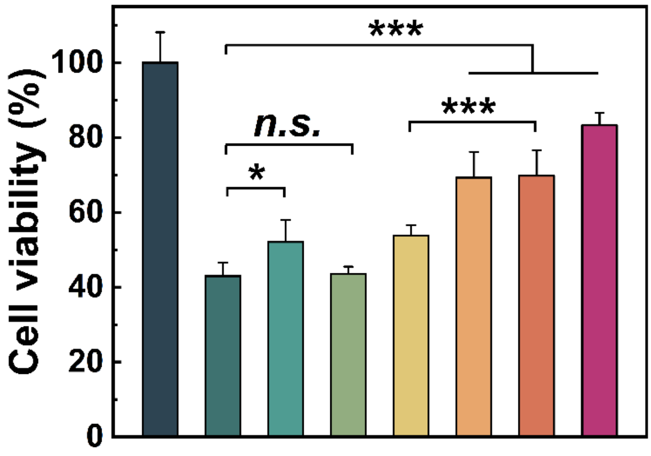

Figure S9: Cell viability of H_2O_2-induced acute injured HK-2 cells after 12 h treatment.

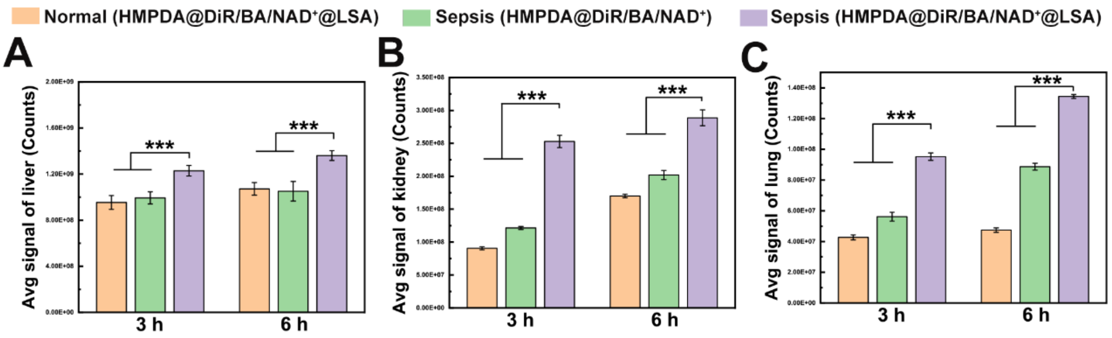

Figure S10: Mean fluorescence values in the liver, kidney, and lung of sepsis mice.

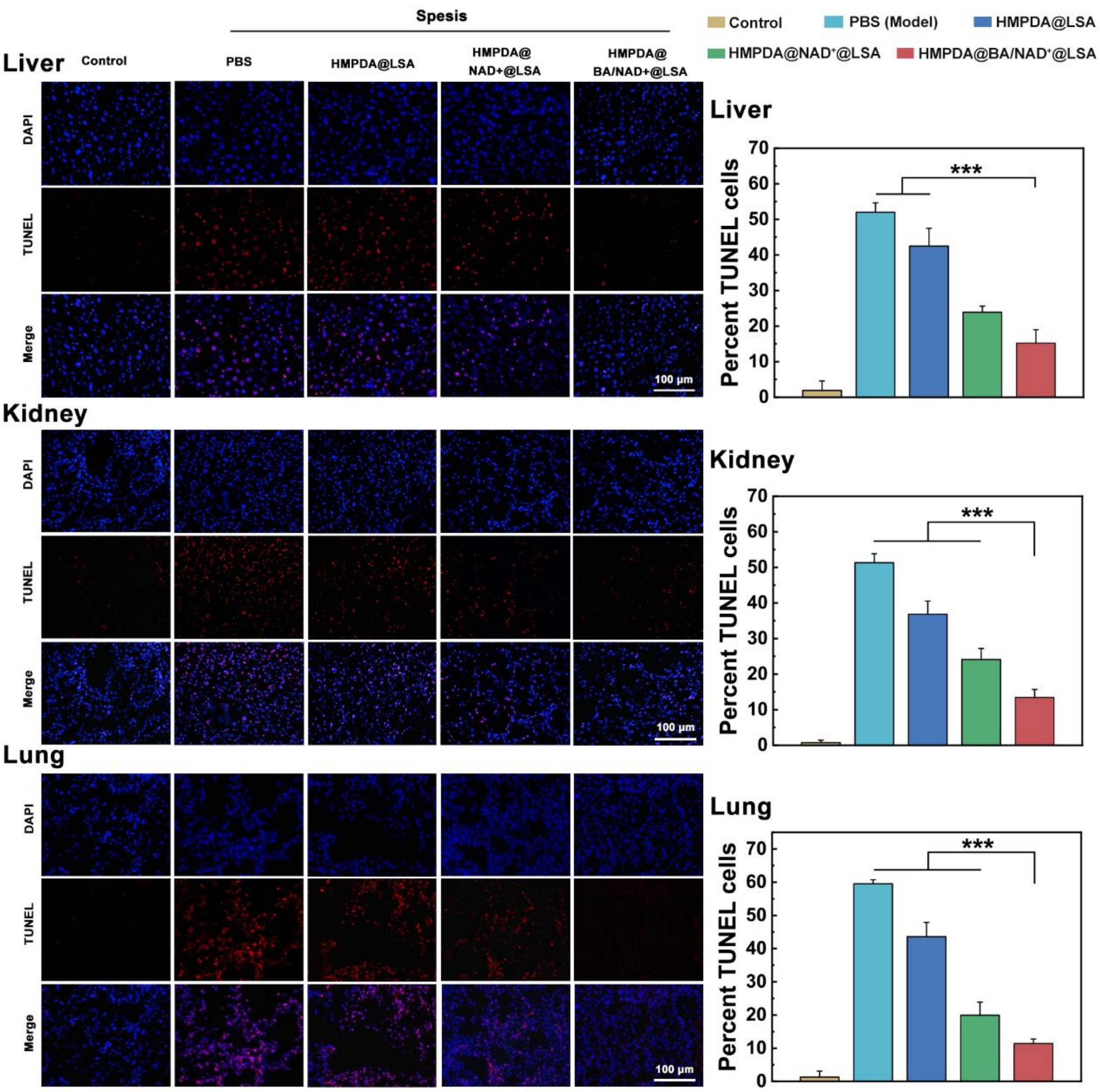

Figure S11: TUNEL staining and semi-quantitative data of liver, kidney, and lung tissus (red fluorescence: TUNEL positive cells; blue fluorescence: cell nucleus).

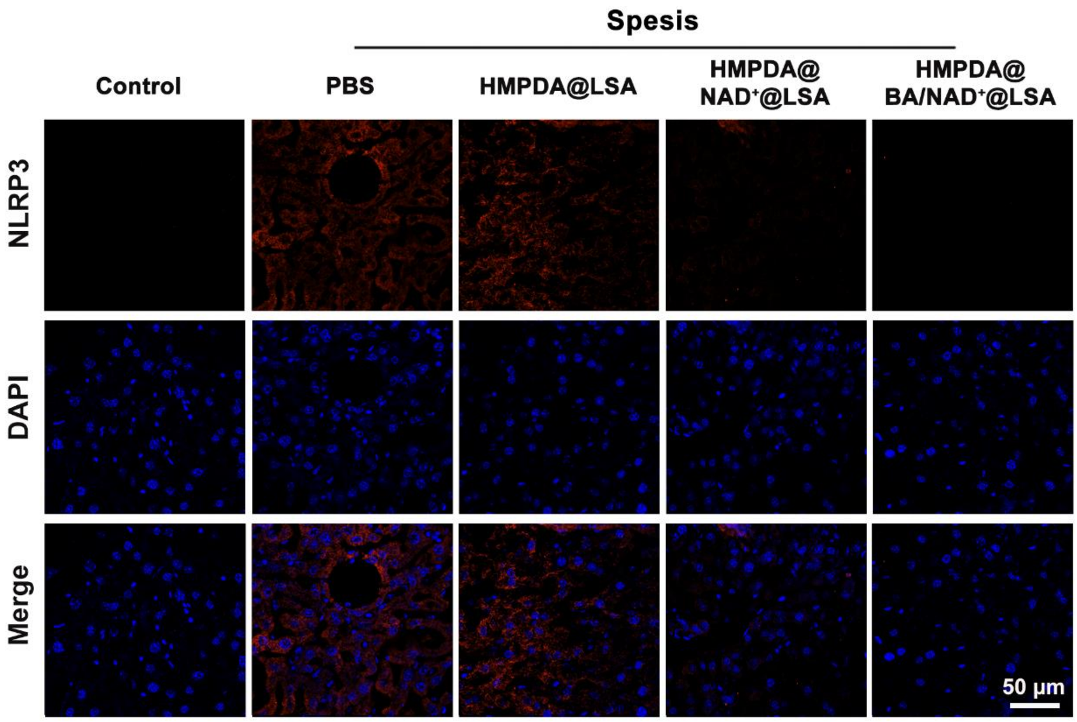

Figure S12: Immunofluorescent staining of NLRP3 of liver.

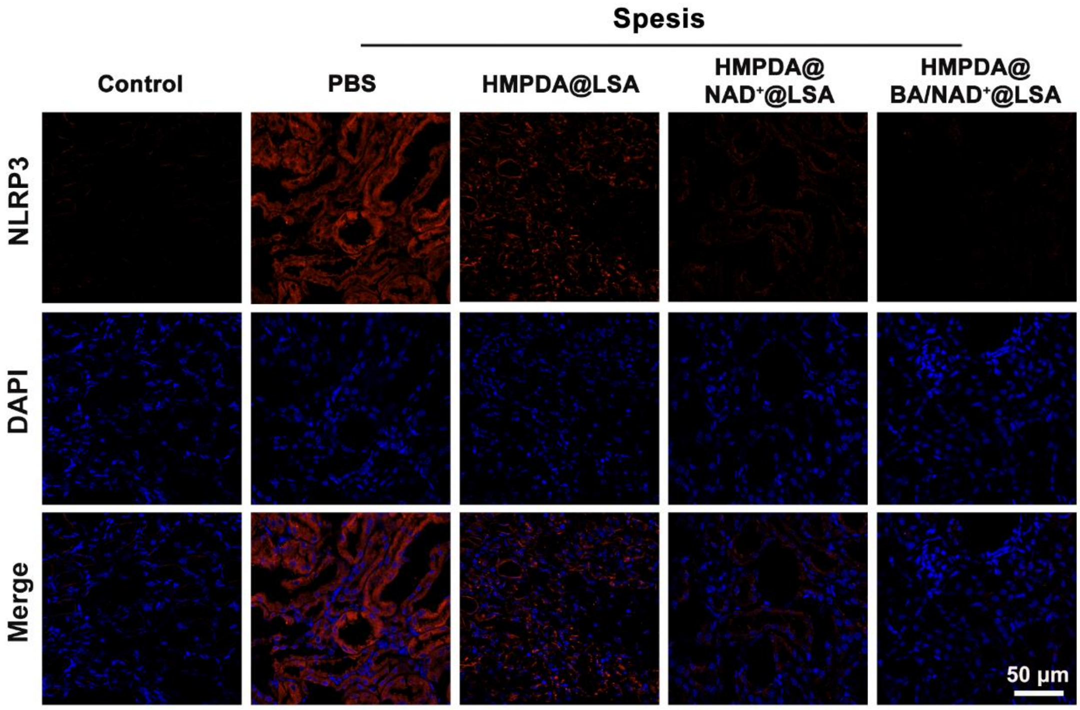

Figure S13: Immunofluorescent staining of NLRP3 of kidney.

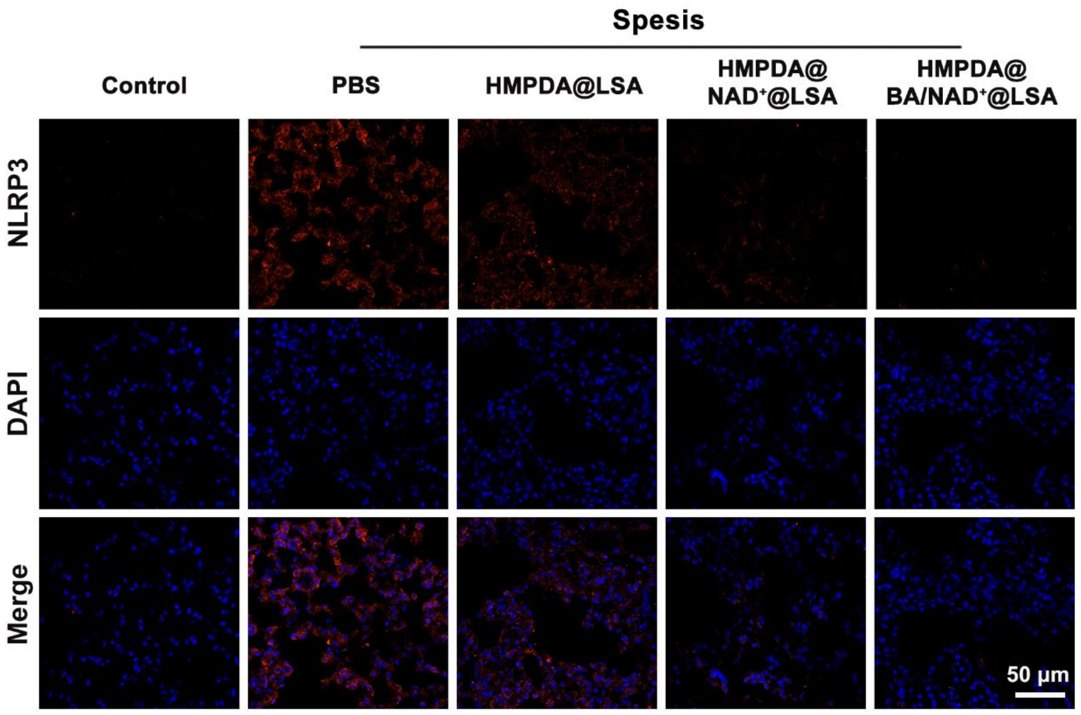

Figure S14: Immunofluorescent staining of NLRP3 of lung.

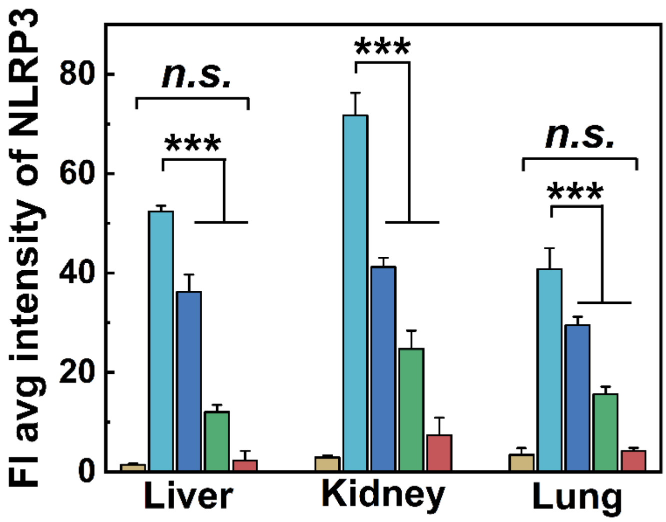

Figure S15: Semi-quantitative of immunofluorescent results of NLRP3.

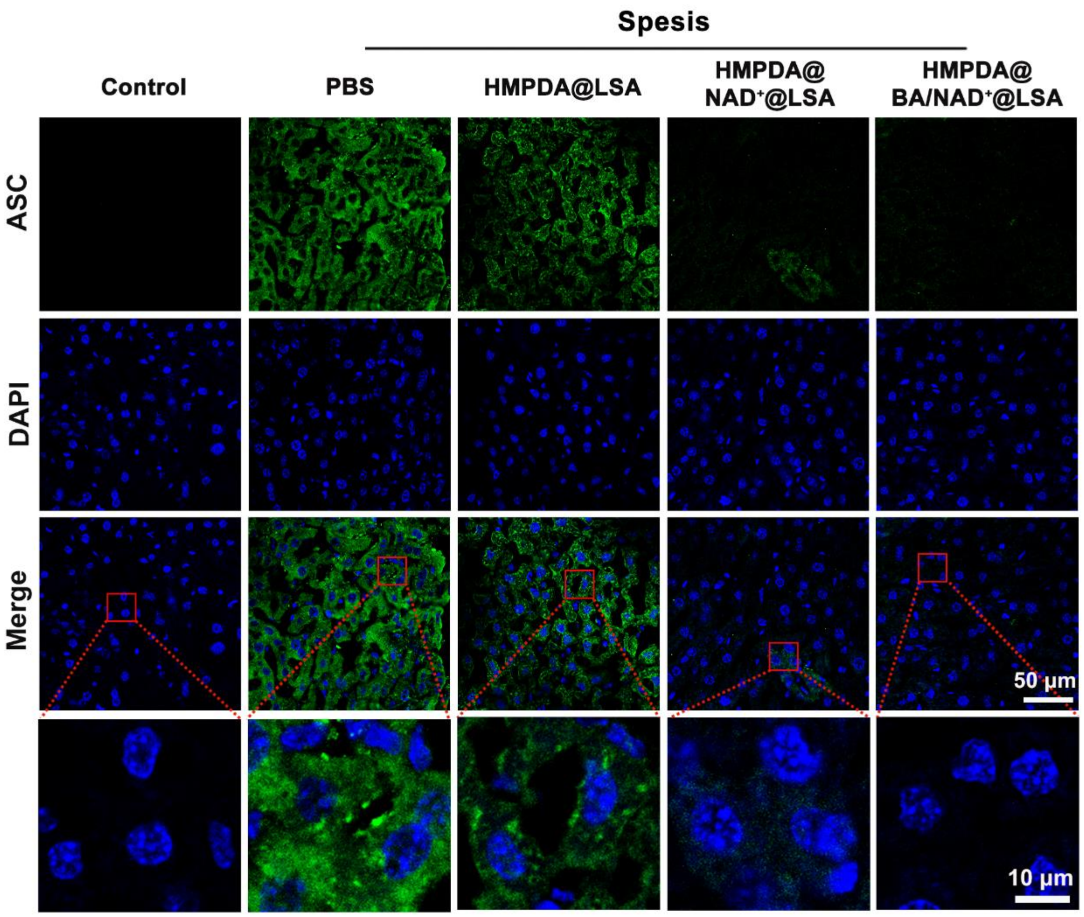

Figure S16: Immunofluorescent staining of ASC of liver.

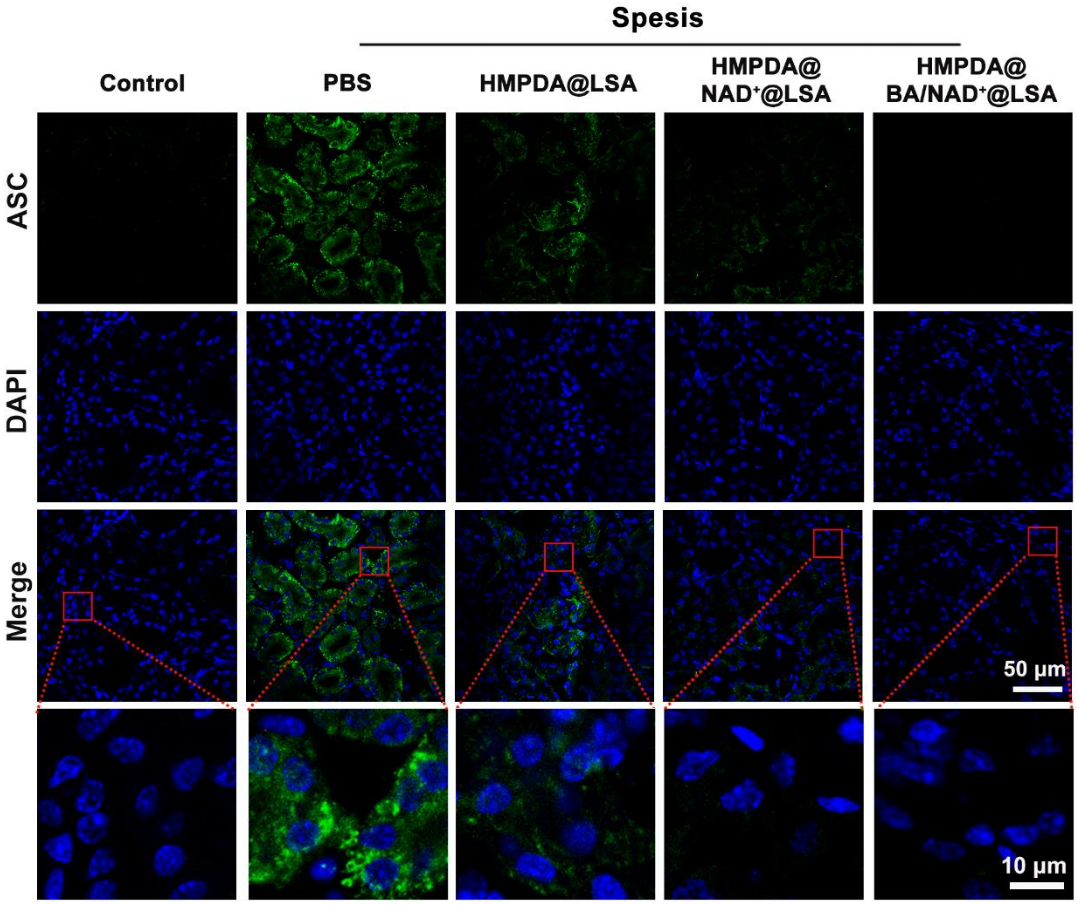

Figure S17: Immunofluorescent staining of ASC of kidney.

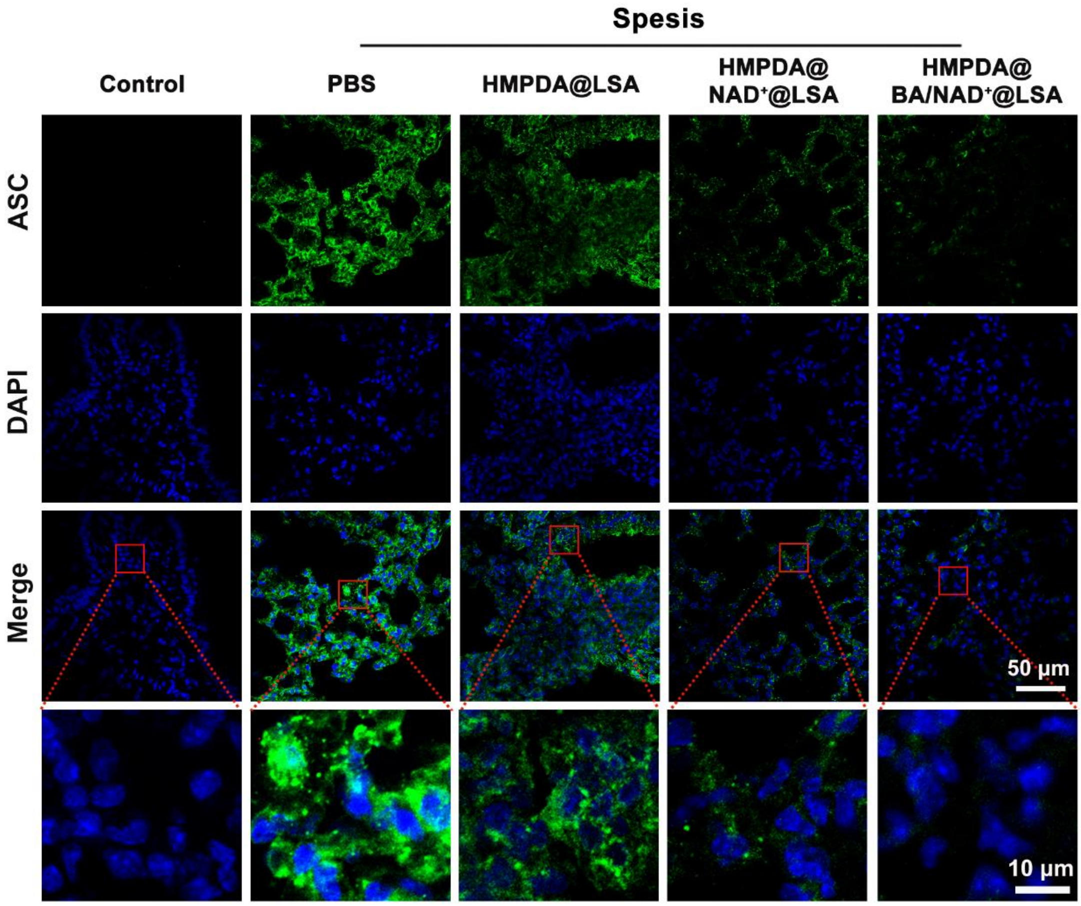

Figure S18: Immunofluorescent staining of ASC of lung.

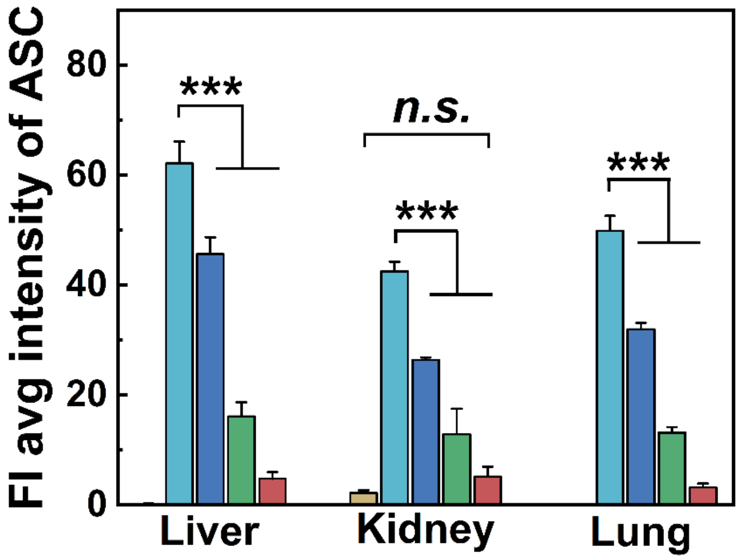

Figure S19: Semi-quantitative of immunofluorescent results of ASC.

Data are mean ± SD, n = 3.

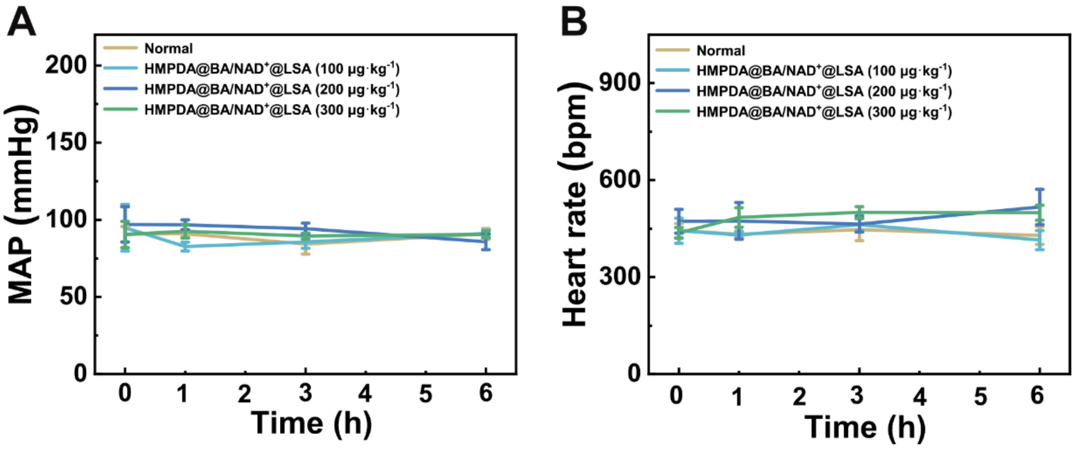

Figure S20: (a) MAP and (b) heart rate in healthy mice. Data are mean ± SD, n = 3.
